# 1,3,5-Triaza-7-Phosphaadamantane (PTA) as a ^31^P NMR Probe for Organometallic Transition Metal Complexes in Solution

**DOI:** 10.3390/molecules26051390

**Published:** 2021-03-04

**Authors:** Ilya G. Shenderovich

**Affiliations:** Institute of Organic Chemistry, University of Regensburg, Universitaetstrasse 31, 93053 Regensburg, Germany; Ilya.Shenderovich@ur.de

**Keywords:** solvent effect, ^31^P NMR, condensed matter, polarizable continuum model, reaction field, external electric field

## Abstract

Due to the rigid structure of 1,3,5-triaza-7-phosphaadamantane (PTA), its ^31^P chemical shift solely depends on non-covalent interactions in which the molecule is involved. The maximum range of change caused by the most common of these, hydrogen bonding, is only 6 ppm, because the active site is one of the PTA nitrogen atoms. In contrast, when the PTA phosphorus atom is coordinated to a metal, the range of change exceeds 100 ppm. This feature can be used to support or reject specific structural models of organometallic transition metal complexes in solution by comparing the experimental and Density Functional Theory (DFT) calculated values of this ^31^P chemical shift. This approach has been tested on a variety of the metals of groups 8–12 and molecular structures. General recommendations for appropriate basis sets are reported.

## 1. Introduction

**Figure 1:**
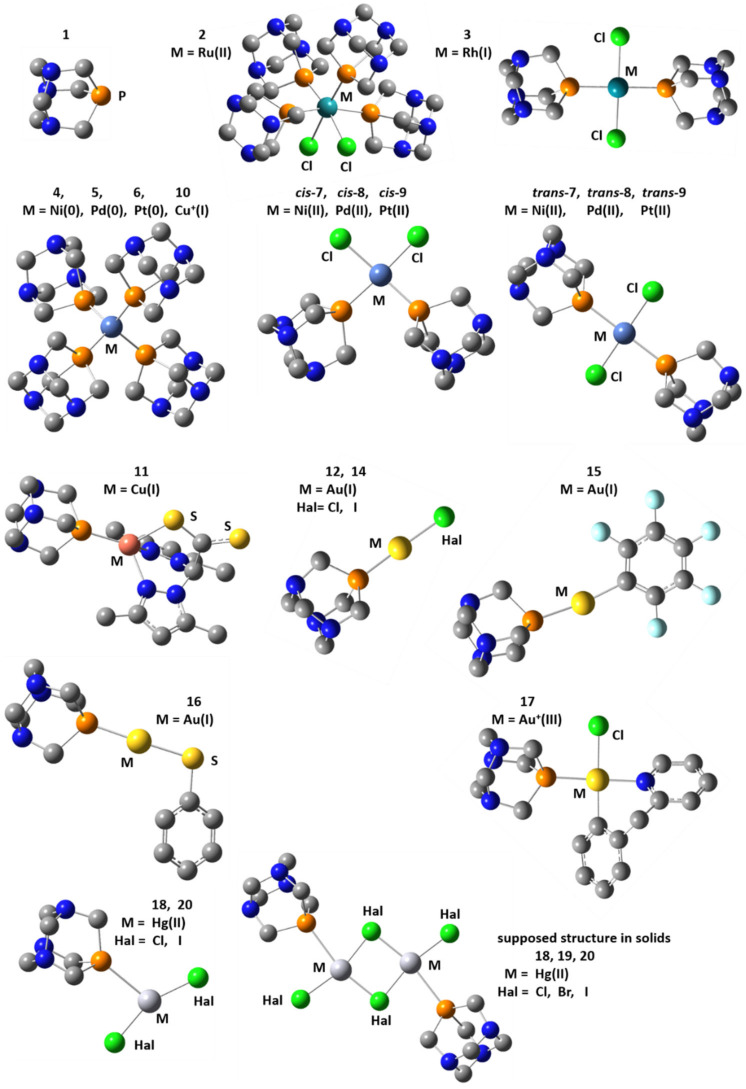
Transition metal complexes of 1,3,5-triaza-7-phosphaadamantane (PTA) studied in this work: 1,3,5-triaza-7-phosphaadamantane (PTA, **1**), *cis*-Cl_2_Ru(PTA)_4_ (**2**), *trans*-Cl_2_Rh(PTA)_2_H (**3**), M(PTA)_4_ (M = Ni (**4**), Pd (**5**), Pt (**6**), Cu+ (**10**)), *cis*-Cl_2_M(PTA)_2_ (M = Ni (*cis-***7**), Pd (*cis-***8**), Pt(*cis-***9**)), *trans*-Cl_2_M(PTA)_2_ (M = Ni (*trans-***7**), Pd (*trans-***8**), Pt(*trans-***9**)), bis(3,5-dimethylpyrazol-1-yl)dithioacetate Cu(I) PTA ([LCS_2_]Cu(PTA), **11**), HalAu(PTA) (Hal = Cl (**12**), I (**14**)), F_5_C_6_Au(PTA) (**15**), H_5_C_6_SAu(PTA) (**16**), [(py^b^-H)ClAu]^+^(PTA) (py^b^-H = C^N cyclometallated 2-benzylpyridine, **17**), Hal_2_Hg(PTA) (Hal = Cl (**18**), Br (**19**), I (**20**)).

**Figure 2:**
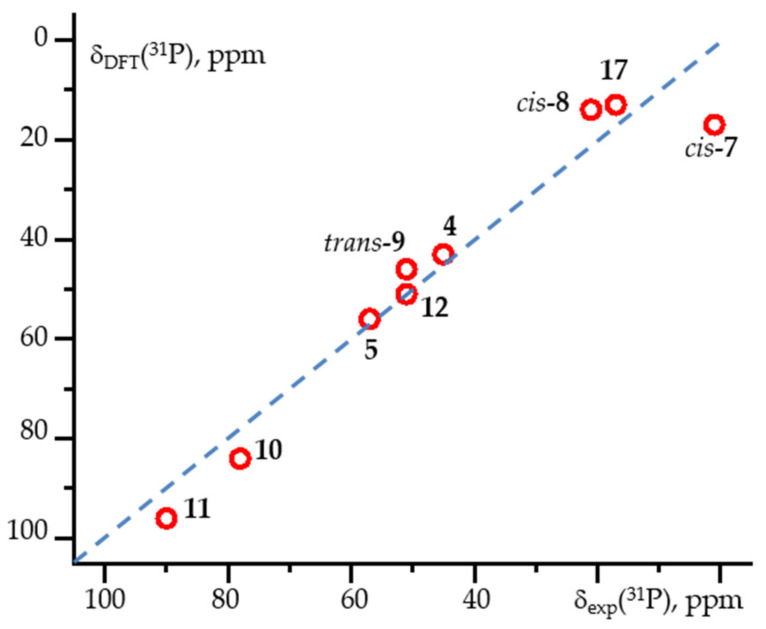
Calculated isotropic ^31^P NMR chemical shift, δ_DFT_(^31^P), compared to experimentally reported values, δ_exp_(^31^P), for selected complexes.

Transition metal organometallics are critically important in modern chemistry, regardless of whether it concerns novel reaction pathways or enhanced selectivity. The exact chemical composition, structure and conformation of these catalysts may be very complex and dependent on their environment [1,2,3,4,5,6]. Therefore, structures present in the crystalline state need not necessarily be close to those in solution. Nowadays, NMR spectroscopy represents the most versatile technique for the elucidation of structures in solution. While ^1^H and ^13^C NMR data are not always sufficient to determine the structure of organometallic species, ^15^N and ^31^P NMR can be very useful when organic ligands are coordinated to the metal center through nitrogen or phosphorus atoms. The efficiency of these methods obviously depends on the magnitude of the chemical shift change caused by the interaction. This magnitude depends on the chemical structure of the ligand. Chemical shift is a tensor quantity, the components of which are δ_11_, δ_22_, and δ_33_. In solution NMR, this anisotropy is averaged out by fast molecular tumbling, and only a single isotropic chemical shift value is observed, δ_iso_ = (δ_11_ + δ_22_ + δ_33_)/3. Therefore, the range of δ_iso_ values does not depend on these values themselves, but on the cumulative range. Even when the change of δ_11_, δ_22_, and δ_33_ is large, the change of δ_iso_ can be small. A number of molecular structures are known in which intermolecular interactions cause large changes in δ_iso_ that increase monotonically with increasing interaction strength. Among such structures is nitrogen heterocycles [6]. These molecules have been successfully used in the past to study intermolecular interactions in crystals [7,8,9], solution [10,11,12], interfaces [13,14,15], and transition metal organometallics [16,17]. The main shortcoming of ^15^N NMR is its low sensitivity. This flaw is absent in ^31^P NMR. The ^31^P isotope is present in 100% natural abundance, has a spin quantum number of 1/2, as well as a chemical shift range of more than 400 ppm. ^31^P NMR can be routinely used in the evaluation of the structure of organic complexes in solution [18,19], interfaces [20,21,22], and solids [23,24] including organometallic compounds [17,25,26]. Considerable progress has been achieved in recent years in our understanding of the effects of non-covalent interactions on the ^31^P chemical shift of P=O groups [25,27,28]. However, there are a number of limitations here too. P=O groups can form two non-covalent interactions simultaneously [29,30,31]. Therefore, the structure of organometallic compounds in solution can be highly dependent on competing solute–solvent interactions. Because the phosphorus atom of the P=O group is not directly involved in intermolecular interactions, its chemical shift is not strictly specific to a certain interaction, and different structures can result in similar chemical shift values [32]. On the contrary, when the observed nucleus is directly involved in the interaction, NMR allows very accurate analysis [33]. ^31^P NMR chemical shift may depend on the conformation of the attached groups [25]. This effect is absent for rigid molecules, such as 1,3,5-triaza-7-phosphaadamantane (PTA, Figure 1) and 2,8,9-trioxa-1-phosphadamantane [34]. PTA is especially useful as an NMR probe because its phosphorus atom is chemically inert. Its ^31^P NMR chemical shift in the crystalline phase can be reproduced correctly in single molecule Density Functional Theory (DFT) calculations [34] without the need to use the gauge-including projector augmented wave (GIPAW) approach [35,36]. In acidic solution, PTA would be protonated at one of its nitrogen atoms. This protonation results in a 6 ppm change in the ^31^P NMR chemical shift [37]. In contrast, when PTA is coordinated to transition metals, its chemical shift varies in a wide range [38]. For some complexes, the value is close to that of the oxidized, P=O form of PTA [39]. It can be expected that due to the symmetry of PTA, its ^31^P NMR shielding tensor in these transition metal complexes remains mainly axisymmetric; that is, two tensor components are lying in a plane normal to the P–metal bond, and their values are similar. In this case, the isotropic chemical shift does not change when PTA rotates around this bond. Consequently, the experimentally measured value of the ^31^P NMR chemical shift of a PTA transition metal complex should be similar to that calculated at the appropriate level of approximation. Of course, the calculated value may deviate from the experimental one due to the presence of a solvent-generated electric field and solute–solvent interactions [39,40,41]. This is the next order of approximation.

This work reports on a computational study of the isotropic ^31^P NMR chemical shift in transition metal complexes of PTA, for which the experimental values of this shift are known. The main objective is to study whether the levels of approximation required for the correct calculation of the ^31^P NMR chemical shift of PTA and its oxidized, P=O form are sufficient for the correct calculation of the ^31^P NMR chemical shift of the transition metal complexes of PTA (Figure 1). These simple calculations can be very helpful. Consider a transition metal complex in which the metal is coordinated with a phosphorus atom. In most cases, the structure of this complex is determined from X-ray diffraction (XRD), while the isotropic ^31^P NMR chemical shift can only be measured in solution. If this value can be reproduced in calculations using the structure of the crystalline phase, then the transition to solution has no significant effect on the structure. If this is not the case, then the experimental ^31^P NMR chemical shift is either not related to the proposed structure, or the structure is labile.

## 2. Results and Discussion

Among the main spectral characteristics of NMR is the absolute chemical shielding, σ. In experiments, σ is not measured directly, but relative to the absolute chemical shielding of a reference, σ_ref_. This relative value is called the chemical shift, δ = (σ^ref^ − σ)/(1 − σ^ref^). σ^ref^ is of the order of 10^−4^, that is, δ ≈ (σ^ref^ − σ). In ^31^P NMR, δ ≡ 0 ppm for 85% H_3_PO_4_ in H_2_O. This value is easy to measure but not to calculate. Recently, the reference values of the absolute chemical shielding, σ^ref^(^31^P), have been reported for a number of approximations [34]. PTA was among the used model compounds.

The values of σ^ref^(^31^P) calculated under the *w*B97XD/Def2QZVP and *w*B97XD/Def2TZVP approximations using PTA as the reference compound are σ^ref^(*w*B97XD/Def2QZVP) = 308 ppm and σ^ref^(*w*B97XD/Def2TZVP) = 306 ppm; these values are valid under the PCM approximations as well (Table S1, Supplementary Materials). The expected margin of error is ±5 and ±9 ppm, respectively [34]. In this paper, the calculated isotropic values of the absolute chemical shielding were converted into the isotropic chemical shifts to compare the calculated values with experimental data, δ_iso_ = (σ^ref^ − σ_iso_). The original absolute chemical shielding tensors are reported in Tables S2 and S3 (Supplementary Materials).

The experimentally reported ^31^P NMR chemical shifts of the transition metal complexes of PTA studied in this work are listed in Table 1. For some of these complexes, their crystal structures are known. However, these structures can change when in solution. The following nomenclature is used in this paper for the structures in calculations. A structure in which the positions of heavy atoms was adapted from the experimental XRD data, while the positions of hydrogen atoms were DFT optimized, is labeled as **n**_XRD_, where **n** is the number of the structure in Table 1. A structure in which the positions of all atoms were DFT optimized is labeled as **n**_opt_. Additionally, all other structures discussed below were optimized with DFT. The calculated ^31^P NMR chemical shifts are listed in Table 2.

### 2.1. PTA Complexes of Group 8 and 9 Transition Metals

Structure **2**_XRD_ was adapted from the experimental structure WELCAR [42]. For this structure, the ^31^P chemical shift was calculated for the Def2TZVP basis set only, because the use of the Def2QZVP basis set results in a convergence problem. This complex contains two sets of chemically nonequivalent PTA molecules (Table 2). This result qualitatively agrees with the experimental data reported for complexes ClHRu(PTA)_4_ and H_2_Ru(PTA)_4_ [42]. The chemical shifts within the same complex can be averaged out in solution. The mean value of δ(^31^P) in **2**_XRD_ is about 5 ppm. This value deviates from the experimental one of −47 ppm. The deviation from the experimental value is also large for the mean δ(^31^P) = −3 ppm in **2**_opt_. The result does not significantly depend on the basis set (Table 2). The fact that in structure **2**_opt_ the δ (^31^P) values within the set of chemically equivalent PTA molecules are not equal suggests that the symmetry of this structure can be increased. However, this also means that small changes in the geometry of the complex lead to significant changes in δ(^31^P), but not in the energy of the complex. In solution, the δ(^31^P) values of all available conformations will be averaged to a certain mean value. Therefore, the δ(^31^P) value for the exact global minimum structure does not matter very much in the case under consideration. How strongly does this chemical shift depend on the charge state of Ru? The mean δ (^31^P) in a doubly charged cation Ru^2+^(PTA)_4_ is about 21 ppm.

Consequently, the experimental value of δ (^31^P) could not be reproduced in any of the studied structures of **2**. On the other hand, we see that the calculated values strongly depend on the molecular structure and the charge state. Therefore, the observed deviations are most likely the result of the fact that none of these structures is close to the average structure of this potentially flexible complex in solution, and the neglect of specific interactions with water molecules, and not a failure of the calculation approach.

Structure [**3**_XRD_]^−^ was adapted from the experimental structure YACGAM [43]. One proton, located at one of the PTA nitrogen atoms, was removed. As a result, this complex of Rh(I) was negatively charged. The calculated value was shifted to a high field by about 17 ppm compared to the experimental one. The optimization of the structure resulted in a further increase in the difference, [**3**_opt_]^−^. Removal of both halogen ligands caused a very large high field shift, Rh^+^(PTA)_2_. This situation is similar to complex **2**. The calculated values do not critically deviate from the experimental one but strongly depend on the molecular structure and the charge state.

### 2.2. PTA Complexes of Group 10 Transition Metals

It is reasonable to expect that the effect of the environment on the structure and spectral properties of highly symmetric complexes of M(0)(PTA)_4_ will be small. Indeed, for Ni and Pd, **4**_opt_ and **5**_opt_, the calculated values of the ^31^P NMR chemical shift are very close to the experimental values and follow the experimental trend. In contrast, for Pt, **6**_opt_, the deviation is large, and the trend is opposite (Table 1 and Table 2). The results are very similar for the smaller basis set Def2TZVP (Table 2).

Cl_2_M(II)(PTA)_2_ complexes may have either the *cis*- or *trans*-configuration. The configuration of complex **7** was not reported. The value calculated for structure *cis*-**7**_opt_ is close enough to the experimental value, while for structure *trans*-**7**_opt_, the deviation is much larger. Structure *cis*-**8**_XRD_ was adapted from the experimental structure PUJNAJ [54]. The two PTA molecules are not equivalent in this structure. The corresponding mean value of δ(^31^P) is close to the value in *cis*-**8**_opt_ as well as to the experimental one. For structure *trans*-**8**_opt_, the deviation is much larger. Structure *cis*-**9**_XRD_ was adapted from the experimental structure JUSQUJ [55]. The deviation from the experimental value is large and increased after the optimization of the structure, *cis*-**9**_opt_. Structure *trans*-**9**_XRD_ was adapted from the experimental structure NOKQIP [56]. The value of δ(^31^P) calculated for this structure is close to both the experimental one and to the value calculated for *trans*-**9**_opt_. Note that for **8** and **9**, the experimental values in water and dimethyl sulfoxide (DMSO) are similar (Table 1).

The calculated values of δ(^31^P) for the PTA complexes of group 10 transition metals agree with the experimental ones for Ni and Pd in both studied types of complexes. For the complex of Pt(II), the calculated value agrees with the experimental one, assuming, in in contradistinction to [45,46], that this complex adopts the *trans*-configuration in solution. The deviation obtained for complex **6** cannot be explained with certainty. Presumably, structure **6**_opt_ does not correspond to the averaged structure of **6** in solution. Whether the Pt-P distances are longer than those optimized by DFT, or the symmetry of the complex differs from the tetrahedral point group T_d_, this requires a separate study. Within the framework of this paper, it is important that there are no indications that δ(^31^P) cannot be correctly calculated for Pt complexes.

### 2.3. PTA Complexes of Group 11 Transition Metals

The calculated values of δ(^31^P) in Cu(I) complexes **10**_opt_^+^ and **11**_opt_ agree with the experimental values (Table 1 and Table 2).

For Au(I) complexes **12**_XRD_ and **12**_opt_, the difference between the calculated and experimental values is about 10 ppm. Structure **12**_XRD_ was adapted from the experimental structure YIKHOP [51]. The deviation increased further for complex **14**_opt_. There was no reason to expect that the result would be better for **13**. Although this deviation can be attributed to a fast exchange with the cation Au^+^(PTA) (Table 2), the presence of this cation in the organic solvent is hard to justify. Instead, the situation could be similar to other complexes in which halogens interact with solvent molecules. Such interactions cause considerable geometric and spectral changes [41,57]. These changes can be modeled using the Adduct under Field (AuF) approach [40] explained in the Materials and Methods section. The expected strength of the field should be smaller than 0.0200 a.u. [57]. Under the effect of the external field, the structure of complex **12** changed somewhat. The value of δ(^31^P) changed slowly and became close to the experimental one when the field strength was about 0.0075-0.0100. a.u. (Table 3). The same trend is expected for complexes **13** and **14**. Therefore, for complexes **12**–**14**, the deviations between the experimental values and the values calculated in the absence of the external electric field should be attributed to the effect of the solvation of the halogen atom. Note that this effect has been reported in the past for other systems as well [58].

For Au(I) complexes **15** and **16**, the difference between the calculated and experimental values is large. Structures **15**_XRD_ and **16**_XRD_ were adapted from the experimental structures VEGCIW [50] and ZIRCAI [51]. The optimization of these structures did not lead to improvements in **15**_opt_ and **16**_opt_ (Table 2). An external electric field caused an increase in the Au–PTA distance. In **15**, it changed from 2.30 Å in the absence of the field to 2.32 Å at the field strength of 0.0075 a.u. However, these changes did not lead to changes of δ(^31^P) (Table 3). Presumably, the structures and/or compositions of these complexes in solution deviate from those in crystals and isolated adducts.

The calculated values of δ(^31^P) in Au(III) complex **17**_XRD_ agree with the experimental values. The optimization of this structure did not lead to a significant change in **17**_opt_.

### 2.4. PTA Complexes of Group 12 Transition Metals

Neither the known structures of the PTA complexes of group 12 transition metals nor ^31^P NMR data were found for such complexes in solution. The δ(^31^P) value of Hg (II) complexes **18**-**20** was measured in solids [53]. It was suggested that the structural units of these solids are centrosymmetric adducts in which two mercury atoms share two halogen atoms and are additionally coordinated to one halogen atom and one PTA molecule each (Figure 1). Such units are difficult to model in calculations. Instead, δ(^31^P) was calculated for structures **18**_opt_(gas), **20**_opt_(gas), **18**_opt_(water), **20**_opt_(water), and Hg^2+^(PTA) without and under the PCM approximation (Table 2). None of these structures can be considered a good model of the experimental structure. Therefore, it is not surprising that the calculated values deviate from the experimental ones. However, the calculated values of δ(^31^P) are sufficient to justify the following qualitative conclusions: (i) the δ(^31^P) of the phosphorus-based ligand of group 12 transition metal complexes can be correctly calculated using conventional DFT calculations; (ii) these quantities should be calculated under the PCM approximation even for solids.

## 3. Methods

The Gaussian 09.D.01 program package was used (Gaussian, Inc., Wallingford, USA) [59]. Geometry optimizations were performed under the *w*B97XD/Def2TZVP approximation [60,61]. The default SCRF=PCM method was used to construct the solute cavity. Unless otherwise indicated, calculations were performed in the solvents used for the corresponding experimental measurements.

For a number of selected molecular systems, the geometry and NMR quantities were calculated using the Adduct under Field (AuF) approach [40]. The macroscopic electric field generated by the dipole moments of the solvent molecules and weak yet multiple interactions with the solvent molecules caused changes in the electron density of the solvated molecules. These changes can be simulated using the external electric field of a selected strength. The external electric field was added to calculations using the keyword “field”. This electric field was directed along the phosphorus–metal bond, so that the energy of the molecular system would decrease. This approach has been successfully used in the past to study dependencies among the properties of electron distribution, molecular properties, and intermolecular interactions [62,63,64,65,66]. Note that this external electric field is fictitious in nature. It is merely a tool to put pressure on the electron density of the molecule under investigation in order to model the changes caused by multiple interactions with solvent molecules. Therefore, this fictitious external electric field should not be confused with real local electric fields present in electrolytes [67] or real external electric fields applied to change the physical [68,69] and chemical [70,71] properties of solutions. At the same time, the strength of this fictitious field is only about one order of magnitude smaller than that of these real electric fields.

There are two known crystal structures of PTA: TAZPAD [72] and TAZPAD02 [73].

## 4. Conclusions

In this work, ^31^P NMR chemical shifts in 19 transition metal complexes of PTA were calculated using static DFT methods. These calculated values were compared with the available experimental values. The most important conclusions are as follows.

The *w*B97XD/Def2QZVP level of approximation is sufficient to correctly calculate the ^31^P NMR chemical shift of phosphorus-based ligand transition metal complexes of groups 8–12 in solution. The Def2TZVP basis set can be used as well. A slight decrease in the accuracy of calculations was offset by a sharp decrease in the calculation costs (Tables S2 and S3, Supplementary Materials).

The calculations revealed the configuration of complexes **7**-**9** in solution. Complex **7** adopted the *cis*-configuration. Complex **8** was in the *cis*-configuration in both crystal and solution. In contrast, complex **9** adopted the *trans*-configuration in solution, even when crystals of the *cis*-configuration were used to prepare the solution. The accuracy of the calculations could be increased for complexes **12**-**14** by accounting for solvent effects using the AuF approach. The values calculated for these complexes and for complexes **4**, **5**, **10**, **11** and **17** are compared to experimentally reported values in Figure 2.

It seems that the configurations of complexes **2** and **3** were very labile in solution. Therefore, the observed deviations may be the result of the studied structures differing from the average structures of these complexes in solution. The same may be true for complex **6**.

The ^31^P NMR chemical shifts reported for complexes **15** and **16** do not correspond to these complexes.

When calculating NMR chemical shifts in solids, it is necessary to use the PCM approximation. The choice of the dielectric constant value is less important than using this approximation [74].

Although it is not unexpected, it is worth mentioning that the value of the Mulliken charge on the phosphorus atom does not correlate with its ^31^P NMR chemical shift (Tables S2 and S3, Supplementary Materials).

## Figures and Tables

**Table 1:** Experimental ^31^P NMR isotropic chemical shifts of transition metal complexes of PTA.

Complex	Solvent	δ_iso_(^31^P), ppm	References
PTA (**1**)	crystalline	−104.3	[34]
*cis*-Cl_2_Ru(PTA)_4_ (**2**)	water	−47.3	[42]
*trans*-Cl_2_Rh(PTA)_2_H (**3**)	water	−2.4	[43]
Ni(PTA)_4_ (**4**)	water	−44.8, −45.7	[44,45]
Pd(PTA)_4_ (**5**)	water	−56.5, −58.7	[44,45]
Pt(PTA)_4_ (**6**)	water	−74.5	[45]
Cl_2_Ni(PTA)_2_ (**7**)	water	−1.2	[46]
*cis*-Cl_2_Pd(PTA)_2_ (**8**)	water	−21	[45]
*cis*-Cl_2_Pd(PTA)_2_ (**8**)	DMSO	−18	[46]
*cis*-Cl_2_Pt(PTA)_2_ (**9**)	water	−51	[45]
*cis*-Cl_2_Pt(PTA)_2_ (**9**)	DMSO	−47.5	[46]
[NO_3_]^-^ Cu^+^(PTA)_4_ (**10**)	water	−78.2	[47]
[LCS_2_]Cu(PTA) (**11**)	CDCl_3_	−90.2	[48]
ClAu(PTA) (**12**)	DMSO	−51.4	[49]
BrAu(PTA) (**13**)	DMSO	−47.3	[49]
IAu(PTA) (**14**)	DMSO	−47.6	[49]
F_5_C_6_Au(PTA) (**15**)	acetone	−46.8	[50]
H_5_C_6_SAu(PTA) (**16**)	no data	−49.8	[51]
[(py^b^-H)ClAu]^+^(PTA) (**17**)	acetone	−16.6	[52]
Cl_2_Hg(PTA) (**18**)	solid	−38.3	[53]
Br_2_Hg(PTA) (**19**)	solid	−44.1	[53]
I_2_Hg(PTA) (**20**)	solid	−61.4	[53]

**Table 2:** ^31^P NMR isotropic chemical shifts calculated under the *ω*B97XD and PCM approximations.

Structure	Basis	Solvent	δ_iso_(^31^P), ppm
*cis*-Cl_2_Ru(PTA)_4_; **2**_XRD_	Def2TZVP	water	30; 29; −18; −23
*cis*-Cl_2_Ru(PTA)_4_; **2**_opt_	Def2QZVP	water	23; 21; −22; −35
*cis*-Cl_2_Ru(PTA)_4_; **2**_opt_	Def2TZVP	water	29; 26; −17; −31
Ru^2+^(PTA)_4_	Def2QZVP	water	70; 70; −28; −28
[*trans*-Cl_2_Rh(PTA)_2_]^−^; **3**_XRD_^-^	Def2QZVP	water	−19; −19
[*trans*-Cl_2_Rh(PTA)_2_]^−^; **3**_opt_^-^	Def2QZVP	water	−25; −25
Rh^+^(PTA)_2_	Def2QZVP	water	−175; 175
Ni(PTA)_4_; **4**_opt_	Def2QZVP	water	−46; −46; −47; −47
Ni(PTA)_4_; **4**_opt_	Def2TZVP	water	−42; −42; −43; −43
Pd(PTA)_4_; **5**_opt_	Def2QZVP	water	−53; −56; −57; −57
Pd(PTA)_4_; **5**_opt_	Def2TZVP	water	−51; −53; −53; −53
Pt(PTA)_4_; **6**_opt_	Def2QZVP	water	−34; −39; −39; −39
Pt(PTA)_4_; **6**_opt_	Def2TZVP	water	−30; −35; −35; −35
Cl_2_Ni(PTA)_2_; *cis-***7**_opt_	Def2QZVP	water	−13; −21
Cl_2_Ni(PTA)_2_; *trans-***7**_opt_	Def2QZVP	water	−36; −36
Cl_2_Pd(PTA)_2_; *cis-***8**_XRD_	Def2QZVP	water	−15; −24
Cl_2_Pd(PTA)_2_; *cis-***8**_opt_	Def2QZVP	water	−13; −14
Cl_2_Pd(PTA)_2_; *trans-***8**_opt_	Def2QZVP	water	−37; −37
Cl_2_Pt(PTA)_2_; *cis-***9**_XRD_	Def2QZVP	water	−20; −24
Cl_2_Pt(PTA)_2_; *cis-***9**_opt_	Def2QZVP	water	−17; −18
Cl_2_Pt(PTA)_2_; *trans-***9**_XRD_	Def2QZVP	water	−48; −48
Cl_2_Pt(PTA)_2_; *trans-***9**_opt_	Def2QZVP	water	−46; −46
Cu^+^(PTA)_4_; **10**_opt_^+^	Def2QZVP	water	−84; −84; −84; −85
[LCS_2_]Cu(PTA); **11**_opt_	Def2QZVP	CHCl_3_	−96
ClAu(PTA); **12**_XRD_	Def2QZVP	DMSO	−62
ClAu(PTA); **12**_opt_	Def2QZVP	DMSO	−62
IAu(PTA); **14**_opt_	Def2QZVP	DMSO	−70
Au^+^(PTA)	Def2QZVP	DMSO	−26
F_5_C_6_Au(PTA); **15**_XRD_	Def2QZVP	CHCl_3_	−73
F_5_C_6_Au(PTA); **15**_opt_	Def2QZVP	CHCl_3_	−76
H_5_C_6_SAu(PTA); **16**_XRD_	Def2QZVP	CHCl_3_	−78
H_5_C_6_SAu(PTA); **16**_opt_	Def2QZVP	CHCl_3_	−74
[(py^b^-H)ClAu]^+^(PTA); **17**_XRD_	Def2QZVP	acetone	−16
[(py^b^-H)ClAu]^+^(PTA); **17**_opt_	Def2QZVP	acetone	−13
Cl_2_Hg(PTA); **18**_opt_(gas)	Def2QZVP	−	−104
Cl_2_Hg(PTA); **18**_opt_(water)	Def2QZVP	water	−77
I_2_Hg(PTA); **20**_opt_(gas)	Def2QZVP	−	−103
I_2_Hg(PTA); **20**_opt_(water)	Def2QZVP	water	−74
Hg^2+^(PTA)	Def2QZVP	water	−37

**Table 3:** ^31^P NMR isotropic chemical shifts calculated under the *ω*B97XD/ Def2QZVP and PCM approximations in the presence of the external electric field.

Structure	Field, a.u.	Solvent	δ_iso_(^31^P), ppm
ClAu(PTA); **12**_opt_25_	25	DMSO	−62
ClAu(PTA); **12**_opt_50_	50	DMSO	−60
ClAu(PTA); **12**_opt_75_	75	DMSO	−55
ClAu(PTA); **12**_opt_100_	100	DMSO	−46
F_5_C_6_Au(PTA); **15**_opt_25_	25	CHCl_3_	−77
F_5_C_6_Au(PTA); **15**_opt_50_	50	CHCl_3_	−78
F_5_C_6_Au(PTA); **15**_opt_75_	75	CHCl_3_	−78

## Data Availability

No new data were created or analyzed in this study. Data sharing is not applicable to this article.

## Supplementary Materials

The following are available online. Table S1: 31P NMR absolute shielding tensors of PTA calculated under the ωB97XD/Def2QZVP approximation. Table S2: 31P NMR absolute shielding tensors calculated under the ωB97XD/Def2QZVP approximation. Table S3: 31P NMR absolute shielding tensors calculated under the ωB97XD/Def2TZVP approximation. The atomic coordinates (Å) of cis-Cl2Ru(PTA)4, 2XRD; cis-Cl2Ru(PTA)4, 2opt; Ru2+(PTA)4; [trans-Cl2Rh(PTA)2]-, 3XRD-; [trans-Cl2Rh(PTA)2]-, 3opt-; Rh+(PTA)2; Ni(PTA)4, 4opt; Pd(PTA)4, 5opt; Pt(PTA)4, 6opt; Cl2Ni(PTA)2, cis-7opt; Cl2Ni(PTA)2, trans-7opt; Cl2Pd(PTA)2, cis-8XRD; Cl2Pd(PTA)2, cis-8opt; Cl2Pd(PTA)2, trans-8opt; Cl2Pt(PTA)2, cis-9XRD; Cl2Pt(PTA)2, cis-9opt; Cl2Pt(PTA)2, trans-9XRD; Cl2Pt(PTA)2, trans-9opt; Cu+(PTA)4, 10opt+; [LCS2]Cu(PTA), 11opt; ClAu(PTA), 12XRD; ClAu(PTA), 12opt; IAu(PTA), 14opt; Au+(PTA); F5C6Au(PTA), 15XRD; F5C6Au(PTA), 15opt; H5C6SAu(PTA); 16XRD; H5C6SAu(PTA); 16opt; [(pyb-H)ClAu]+(PTA), **17**XRD; [(py^b^-H)ClAu]^+^(PTA), **17**opt; Cl2Hg(PTA), **18**opt(gas); Cl2Hg(PTA), **18**opt(water); I2Hg(PTA), **20**opt(gas); I2Hg(PTA), **20**opt(water); Hg^2+^(PTA).

## Sample Availability

Not available.
