# Supplementary material for: 1,3,5-Triaza-7-Phosphaadamantane (PTA) as a 31P NMR Probe for Organometallic Transition Metal Complexes in Solution
Source: Molecules. 2021 Mar 4;26(5):1390. doi: 10.3390/molecules26051390 (PMC7961616; doi:10.3390/molecules26051390)
Supplement: Supplementary file 1 [file molecules-26-01390-s001.pdf]

Supporting Materials

1,3,5-Triaza-7-Phosphaadamantane (PTA) as a ^{31}P NMR Probe for Organometallic Transition Metal Complexes in Solution

Ilya G. Shenderovich

Institute of Organic Chemistry, University of Regensburg, Universitaetstrasse 31, 93053 Regensburg, Germany; Ilya.Shenderovich@ur.de

Section 1.

Chemical shift is a tensor quantity, the components of which are $\delta_{11} \geq \delta_{22} \geq \delta_{33}$. In solution NMR, this anisotropy is averaged out by fast molecular tumbling, and only a single isotropic chemical shift value is observed, $\delta_{iso} = (\delta_{11} + \delta_{22} + \delta_{33})/3$. Theoretical calculations provide the absolute chemical shielding tensor, the components of which are $\sigma_{11} \leq \sigma_{22} \leq \sigma_{33}$. $\sigma_{iso} = (\sigma_{11} + \sigma_{22} + \sigma_{33})/3$. $\delta_{iso} = (\sigma^{ref} - \sigma_{iso})$.

Table S1. ^{31}P NMR absolute shielding tensors of PTA calculated under the *ω*B97XD/Def2QZVP approximation.

Basis	PCM Solvent	σ_{iso}, ppm	σ_{11}, ppm	σ_{22}, ppm	σ_{33}, ppm
Def2TZVP	---	410	403	414	414
Def2QZVP	---	412	408	415	415
Def2QZVP	Toluene	411	406	414	414
Def2QZVP	DMSO	410	404	413	413
Def2QZVP	Water	410	404	413	413

The experimental value of the ^{31}P chemical shift in crystalline PTA is δ_{iso} = -104.3 ppm [1].

[1] Shenderovich, I. G. Experimentally Established Benchmark Calculations of ^{31}P NMR Quantities. *Chemistry –Methods* **2021**, *1*, 61–70, DOI: 10.1002/cmtd.202000033.

Table S2. ^{31}P NMR absolute shielding tensors calculated under the *ω*B97XD/Def2QZVP approximation.

Structure	PCM Solvent	σ_{iso}, ppm	σ_{11}, ppm	σ_{22}, ppm	σ_{33}, ppm	q[a]
cis-$Cl_2Ru(PTA)_4$; **2**$_{opt}$	water	287	204	226	431	0.054101
		330	283	292	416	-0.543649
		285	199	221	434	-0.074485
		343	297	319	414	-0.346663
$Ru^{2+}(PTA)_4$	water	238	126	170	418	-0.222149
		336	291	312	405	-0.290324
		238	126	170	418	-0.222089
		336	291	312	405	-0.290186
[*trans*-$Cl_2Rh(PTA)_2]^-$; **3**$_{XRD}^-$	water	327	264	299	418	0.033485
		327	264	299	418	0.033517
[*trans*-$Cl_2Rh(PTA)_2]^-$; **3**$_{opt}^-$	water	333	269	304	425	0.050166
		333	269	304	425	0.050168
$Rh^+(PTA)_2$	water	483	352	354	743	-0.007600
		483	352	354	743	-0.007600
$Ni(PTA)_4$; **4** $_{opt}$	water	354	306	308	450	-0.107370
		354	308	309	446	-0.096343
		355	306	311	449	-0.121543
		355	308	308	449	-0.141728
$Pd(PTA)_4$; **5** $_{opt}$	water	364	327	328	436	-0.170936
		361	323	324	437	-0.189688
		365	327	331	436	-0.173531
		365	328	330	436	-0.182472
$Pt(PTA)_4$; **6**$_{opt}$	water	347	297	301	443	-0.106119
		342	288	289	448	-0.073946
		347	296	300	443	-0.098733
		347	296	301	443	-0.105800
$Cl_2Ni(PTA)_2$; *cis*-**7**$_{opt}$	water	321	258	301	404	0.108029
		329	278	302	406	0.063692
$Cl_2Ni(PTA)_2$; *trans*-**7**$_{opt}$	water	344	312	324	397	0.206707
		344	312	324	397	0.206707
$Cl_2Pd(PTA)_2$; *cis*-**8**$_{XRD}$	water	332	292	297	407	0.110805
		323	270	294	404	0.144806
$Cl_2Pd(PTA)_2$; *cis*-**8**$_{opt}$	water	322	271	290	405	0.167048
		321	264	290	407	0.134686
$Cl_2Pd(PTA)_2$; *trans*-**8**$_{opt}$	water	345	316	318	402	0.146598
		345	316	318	402	0.146598
$Cl_2Pt(PTA)_2$; *cis*-**9**$_{XRD}$	water	328	272	301	413	0.131217
		332	280	302	413	0.103384
$Cl_2Pt(PTA)_2$; *cis*-**9**$_{opt}$	water	325	266	294	414	0.133139
		326	272	296	411	0.141407
$Cl_2Pt(PTA)_2$; *trans*-**9**$_{XRD}$	water	356	324	333	412	0.262388
		356	324	333	412	0.262388
$Cl_2Pt(PTA)_2$; *trans*-**9**$_{opt}$	water	354	325	331	406	0.203431
		354	325	331	406	0.203431
$Cu^+(PTA)_4$; **10**$_{opt}^+$	water	392	381	385	411	-0.153649
		392	382	383	410	-0.080036
		392	381	385	411	-0.153224
		393	383	384	411	-0.149503
$[LCS_2]Cu(PTA)$; **11**$_{opt}$	$CHCl_3$	404	393	400	419	
ClAu(PTA); **12**$_{XRD}$	DMSO	370	346	356	409	-0.114594

ClAu(PTA); **12**opt	DMSO	370	352	353	406	-0.099675
IAu(PTA); **14**opt	DMSO	378	364	364	406	-0.101854
Au^{+}(PTA)	DMSO	334	302	302	397	-0.147841
F_5C_6Au(PTA); **15**XRD	$CHCl_3$	381	364	370	408	0.037136
F_5C_6Au(PTA); **15**opt	$CHCl_3$	384	370	372	409	
H_5C_6SAu(PTA); **16**XRD	$CHCl_3$	386	367	378	412	-0.059768
H_5C_6SAu(PTA); **16**opt	$CHCl_3$	382	368	370	409	-0.053475
[(py[b]-H)ClAu]$^{+}$(PTA); **17**XRD	acetone	324	286	298	388	-0.075234
[(py[b]-H)ClAu]$^{+}$(PTA); **17**opt	acetone	321	281	292	391	-0.073127
Cl_2Hg(PTA); **18**opt(gas)	-----	412	404	413	419	-0.011421
Cl_2Hg(PTA); **18**opt(water)	water	385	379	382	394	0.033021
I_2Hg(PTA); **20**opt(gas)	-----	411	406	408	419	0.003446
I_2Hg(PTA); **20**opt(water)	water	382	370	380	395	0.056797
Hg^{2+}(PTA)	water	345	327	327	383	0.174000

[a]The Mulliken charge of the phosphorous atom.

Table S3. ^{31}P NMR absolute shielding tensors calculated under the ωB97XD/Def2TZVP approximation.

Structure	PCM Solvent	σ_{iso}, ppm	σ_{11}, ppm	σ_{22}, ppm	σ_{33}, ppm	q[a]
cis-$Cl_2Ru(PTA)_4$; **2**$_{XRD}$	water	276	188	216	425	0.392638
		324	270	295	407	0.339719
		277	184	219	227	0.370962
		329	278	306	405	0.376701
cis-$Cl_2Ru(PTA)_4$; **2**$_{opt}$	water	280	196	218	425	0.389824
		323	275	284	409	0.339821
		277	190	212	428	0.375025
		337	290	312	407	0.369276
[*trans*-$Cl_2Rh(PTA)_2]^-$; **3**$_{opt}^-$	water	327	263	300	419	0.318457
		327	263	300	419	0.318456
$Ni(PTA)_4$; **4** $_{opt}$	water	348	299	301	443	0.186814
		349	302	303	442	0.178645
		349	300	304	443	0.181922
		348	300	301	443	0.181586
$Pd(PTA)_4$; **5** $_{opt}$	water	359	322	324	430	0.224303
		357	319	320	432	0.303474
		359	321	325	430	0.225690
		359	322	324	430	0.223539
$Pt(PTA)_4$; **6**$_{opt}$	water	341	291	296	437	0.038275
		336	282	283	443	0.135746
		341	291	296	438	0.043332
		341	291	295	437	0.039016
$Cl_2Ni(PTA)_2$; *cis*-**7**$_{opt}$	water	316	253	296	398	0.369638
		323	273	297	399	0.356381
$Cl_2Ni(PTA)_2$; *trans*-**7**$_{opt}$	water	339	306	319	390	0.438339
		339	306	319	390	0.438339
$Cl_2Pd(PTA)_2$; *cis*-**8**$_{opt}$	water	316	265	284	398	0.335398
		315	258	285	400	0.317341
$Cl_2Pd(PTA)_2$; *trans*-**8**$_{opt}$	water	339	310	313	395	0.396654
		339	310	313	395	0.396654
$Cl_2Pt(PTA)_2$; *cis*-**9**$_{opt}$	water	320	262	290	408	0.124906
		321	267	291	404	0.127911
$Cl_2Pt(PTA)_2$; *trans*-**9**$_{opt}$	water	348	319	326	400	0.172353
		248	319	326	400	0.172353
$Cu^+(PTA)_4$; **10**$_{opt}^+$	water	388	378	381	404	0.232643
		386	377	378	404	0.268338
		387	378	381	403	0.232518
		387	378	381	404	0.235990
$[LCS_2]Cu(PTA)$; **11**$_{opt}$	$CHCl_3$	400	390	397	413	0.295766
ClAu(PTA); **12**$_{opt}$	DMSO	367	350	350	400	0.016382
IAu(PTA); **14**$_{opt}$	DMSO	374	361	361	399	0.034889
$F_5C_6Au(PTA)$; **15**$_{opt}$	$CHCl_3$	379	366	369	402	0.088361
$H_5C_6SAu(PTA)$; **16**$_{opt}$	$CHCl_3$	379	366	367	403	0.017321
[(py[b]-H)ClAu]$^+$(PTA); **17**$_{opt}$	acetone	315	274	286	384	0.212631
$Cl_2Hg(PTA)$; **18**$_{opt}$(water)	water	380	375	379	388	0.116434
$I_2Hg(PTA)$; **20**$_{opt}$(water)	water	376	365	375	389	0.149688

[a]The Mulliken charge of the phosphorous atom.

Section 2.

The atomic coordinates (Å) of *cis*-$Cl_2Ru(PTA)_4$; **2**$_{XRD}$.

44	0.012564	0.043208	-0.602827
17	0.135668	-1.765152	-2.307862
17	-0.012299	1.565858	-2.590328
15	-0.250620	1.914972	0.646795
15	-2.295586	-0.323469	-0.851183
15	0.146252	-1.399102	1.122008
15	2.387751	0.042842	-0.858656
7	-1.147805	3.261011	2.924363
7	0.841136	4.216756	1.813721
7	-1.376425	4.445706	0.768985
7	-5.015903	-0.008243	-0.432173
7	-4.298638	-0.315946	-2.773197
7	-4.304731	-2.225938	-1.223015
7	1.566372	-2.455832	3.278083
7	0.343214	-4.068243	1.844668
7	-0.891354	-2.620864	3.425156
7	4.511166	0.548147	-2.591697
7	5.047665	0.558441	-0.186380
7	4.601912	-1.563716	-1.339703
6	-1.011055	1.920455	2.346131
6	1.204038	2.973952	1.087878
6	-1.276069	3.272596	-0.098923
6	0.172472	3.904475	3.076036
6	-0.054962	5.038593	0.985286
6	-1.970358	4.100471	2.060764
6	-3.705231	0.412745	0.097938
6	-2.904721	0.096256	-2.533837
6	-2.889765	-2.079561	-0.804215
6	-4.482557	-1.761656	-2.607725
6	-5.170824	0.366891	-1.846177
6	-5.182957	-1.477201	-0.335143
6	1.591855	-1.363050	2.300522
6	0.215671	-3.188951	0.677981
6	-1.164897	-1.561451	2.442686
6	1.555389	-3.757005	2.606291
6	0.376970	-2.369205	4.121705

6	-0.795803	-3.926580	2.747817
6	3.060031	0.756892	-2.431380
6	3.165580	-1.638386	-1.030881
6	3.660829	0.798392	0.269648
6	5.264738	1.166331	-1.496675
6	4.819185	-0.868575	-2.607363
6	5.324786	-0.871308	-0.278788
1	-0.381804	1.318375	3.005478
1	-1.994402	1.447527	2.306852
1	1.729646	3.242390	0.169252
1	1.883958	2.392153	1.711877
1	-2.274613	2.902397	-0.329982
1	-0.816042	3.547483	-1.045894
1	0.815124	3.254108	3.670796
1	0.022945	4.839370	3.619285
1	0.418356	5.222274	0.020625
1	-0.192418	5.994609	1.494982
1	-2.165327	5.033542	2.594661
1	-2.927400	3.605107	1.887501
1	-3.651623	1.500849	0.062684
1	-3.638147	0.118960	1.148092
1	-2.242667	-0.386221	-3.252411
1	-2.814125	1.171458	-2.683655
1	-2.789408	-2.486061	0.201625
1	-2.251893	-2.661995	-1.467429
1	-3.779052	-2.290625	-3.251029
1	-5.499607	-2.009093	-2.917525
1	-5.017403	1.444150	-1.937003
1	-6.205576	0.149429	-2.122099
1	-6.216864	-1.706916	-0.599749
1	-5.009236	-1.788481	0.695772
1	1.595860	-0.402849	2.821562
1	2.521337	-1.426779	1.737802
1	1.053366	-3.356944	0.001551
1	-0.682923	-3.450602	0.120565
1	-2.131502	-1.754051	1.976732
1	-1.258378	-0.615005	2.973266

1	1.670914	-4.524456	3.374220
1	2.416508	-3.815223	1.937702
1	0.336234	-1.381884	4.584352
1	0.471881	-3.115410	4.913001
1	-0.710923	-4.693581	3.519924
1	-1.718361	-4.101044	2.191487
1	2.837864	1.823463	-2.450642
1	2.528480	0.300237	-3.265551
1	2.640813	-2.163814	-1.825683
1	3.034727	-2.215018	-0.115134
1	3.547511	0.420006	1.285786
1	3.499000	1.874542	0.304818
1	5.009285	2.225697	-1.443712
1	6.327160	1.079168	-1.734652
1	5.875076	-0.980533	-2.867571
1	4.225170	-1.358112	-3.380817
1	5.096848	-1.344673	0.677905
1	6.394240	-0.989402	-0.469585

The atomic coordinates (Å) of *cis*-$Cl_2Ru(PTA)_4$; **2**$_{opt}$.

44	0.044793	0.028597	-0.659727
15	-0.348985	1.824912	0.690659
6	-1.135376	1.667259	2.366233
6	1.023665	2.958319	1.205023
6	-1.471531	3.151136	0.030553
7	-1.347256	2.958109	3.014764
7	0.559203	4.096044	1.995585
7	-1.649874	4.256899	0.967210
6	-2.233975	3.813160	2.228841
6	-0.084507	3.659899	3.231515
6	-0.381289	4.924403	1.245106
1	-2.094654	1.156881	2.271096
1	-0.500499	1.052566	3.006772
1	1.753565	2.393401	1.786298
1	1.517784	3.322984	0.302081
1	-1.038540	3.515164	-0.899685
1	-2.443692	2.724912	-0.219718
1	-2.467068	4.696750	2.825856
1	-3.164157	3.278609	2.027776
1	-0.288152	4.544075	3.838358
1	0.596830	3.009964	3.783077
1	-0.588973	5.819101	1.835165
1	0.078340	5.227585	0.303324
15	-2.255021	-0.365325	-0.982084
6	-3.603549	0.045863	0.233816
6	-3.040106	0.412258	-2.468146
6	-2.789651	-2.110663	-1.324278
7	-4.928465	-0.311323	-0.265751
7	-4.434104	0.008205	-2.638369
7	-4.213827	-2.208273	-1.630426
6	-5.037656	-1.746413	-0.517362
6	-5.251711	0.407582	-1.496978
6	-4.557892	-1.435626	-2.822309
1	-3.424860	-0.479015	1.173234
1	-3.588051	1.113635	0.455385
1	-2.973720	1.496899	-2.377727

1	-2.457426	0.129572	-3.345367
1	-2.198487	-2.478945	-2.161569
1	-2.561021	-2.745192	-0.466317
1	-6.080603	-1.969939	-0.749865
1	-4.760863	-2.291993	0.386545
1	-6.296917	0.205952	-1.739172
1	-5.136176	1.479628	-1.329104
1	-5.595113	-1.657104	-3.081370
1	-3.918257	-1.748394	-3.648824
15	0.177841	-1.478381	1.032677
6	1.433206	-1.243729	2.379515
6	-1.252283	-1.871596	2.158567
6	0.584867	-3.236686	0.605207
7	1.444131	-2.343749	3.339235
7	-0.928437	-2.889929	3.153778
7	0.701656	-4.087118	1.787788
6	1.750500	-3.618359	2.690131
6	0.161368	-2.463229	4.026910
6	-0.556060	-4.154612	2.524421
1	2.425080	-1.144868	1.939111
1	1.230280	-0.306335	2.901462
1	-1.578994	-0.965836	2.669338
1	-2.091229	-2.225948	1.558514
1	-0.196028	-3.619389	-0.053685
1	1.511466	-3.267173	0.034367
1	1.886754	-4.368951	3.470738
1	2.686197	-3.524518	2.136279
1	0.269058	-3.205436	4.819911
1	-0.093870	-1.505786	4.483962
1	-0.452081	-4.904884	3.310408
1	-1.352903	-4.469438	1.848706
15	2.443715	0.154565	-0.829592
6	3.094618	0.758878	-2.457078
6	3.624688	1.113220	0.245184
6	3.380812	-1.453179	-0.795320
7	4.548034	0.651184	-2.558079
7	5.014806	0.959185	-0.179434

7	4.798454	-1.291975	-1.099280
6	4.998625	-0.731735	-2.433593
6	5.215426	1.455931	-1.539528
6	5.457229	-0.430546	-0.121743
1	2.616623	0.177416	-3.245587
1	2.789319	1.796389	-2.586043
1	3.367424	2.170501	0.200999
1	3.523556	0.794359	1.284874
1	3.287710	-1.916531	0.187292
1	2.916377	-2.119558	-1.521980
1	6.066878	-0.763885	-2.657394
1	4.471044	-1.348360	-3.162903
1	6.287487	1.448188	-1.746282
1	4.857010	2.484624	-1.602692
1	6.530876	-0.451383	-0.319015
1	5.284207	-0.824437	0.881328
17	0.379744	-1.751707	-2.423620
17	-0.013417	1.702252	-2.566508

The atomic coordinates (Å) of $Ru^{2+}(PTA)_4$.

15	-0.032167	1.612024	0.840878
6	-1.600099	2.063770	1.719433
6	1.082665	1.623957	2.317806
6	0.384796	3.284952	0.158388
7	-1.417333	3.242818	2.558211
7	0.936246	2.861182	3.076057
7	0.317002	4.302745	1.200741
6	-1.024706	4.410210	1.770058
6	-0.418859	3.016757	3.600209
6	1.271985	4.037740	2.274985
1	-2.379682	2.265744	0.983672
1	-1.935113	1.225074	2.332459
1	0.842235	0.777885	2.963816
1	2.118345	1.507391	2.000171
1	1.389360	3.262614	-0.265658
1	-0.305303	3.528720	-0.650714
1	-1.045474	5.281588	2.425816
1	-1.746573	4.562536	0.966948
1	-0.425914	3.879273	4.267753
1	-0.689076	2.131952	4.177634
1	1.283733	4.903015	2.938887
1	2.268346	3.914129	1.849192
15	-2.366468	-0.029031	-1.045988
6	-3.022531	1.497632	-1.876435
6	-2.802323	-1.240690	-2.384830
6	-3.800722	-0.361930	0.085218
7	-4.384340	1.298352	-2.355885
7	-4.192102	-1.098117	-2.802635
7	-5.056729	-0.320121	-0.652943
6	-5.298403	0.988260	-1.258348
6	-4.458993	0.230200	-3.351671
6	-5.113447	-1.344249	-1.695245
1	-3.000377	2.339573	-1.183122
1	-2.363559	1.745028	-2.710336
1	-2.137365	-1.072871	-3.233760
1	-2.627905	-2.255824	-2.025989

1	-3.688245	-1.346883	0.539721
1	-3.821169	0.374891	0.889228
1	-6.315663	0.994376	-1.652403
1	-5.218412	1.760128	-0.491803
1	-5.467501	0.226852	-3.767942
1	-3.751612	0.437230	-4.155464
1	-6.127941	-1.360345	-2.096146
1	-4.898148	-2.318822	-1.255420
15	0.031454	-1.611051	0.842092
6	-1.083750	-1.621583	2.318728
6	-0.385228	-3.284611	0.161009
6	1.599234	-2.061838	1.721427
7	-0.937434	-2.858091	3.078144
7	-0.317616	-4.301375	1.204354
7	1.416339	-3.240123	2.561255
6	0.417556	-3.013059	3.602768
6	-1.272887	-4.035465	2.278118
6	1.023953	-4.408263	1.774071
1	-0.843318	-0.774940	2.963953
1	-2.119365	-1.505125	2.000803
1	-1.389668	-3.262813	-0.263385
1	0.305183	-3.528966	-0.647654
1	2.379072	-2.264312	0.986071
1	1.933853	-1.222489	2.333774
1	0.424477	-3.874890	4.271197
1	0.687585	-2.127624	4.179314
1	-1.284740	-4.900069	2.942895
1	-2.269166	-3.912348	1.852003
1	1.044621	-5.279006	2.430671
1	1.746016	-4.561343	0.971283
15	2.367009	0.028141	-1.046039
6	2.803937	1.238544	-2.385686
6	3.800776	0.361445	0.085649
6	3.022943	-1.499361	-1.874970
7	4.193897	1.095188	-2.802577
7	5.057178	0.318569	-0.651815
7	4.385095	-1.300987	-2.353812

6	4.460657	-0.233679	-3.350424
6	5.114747	1.341878	-1.694876
6	5.298690	-0.990387	-1.256064
1	2.139405	1.070190	-3.234846
1	2.629531	2.254026	-2.027826
1	3.688429	1.346802	0.539304
1	3.820418	-0.374775	0.890252
1	2.999968	-2.340835	-1.181155
1	2.364356	-1.747007	-2.709111
1	5.469391	-0.230990	-3.766151
1	3.753624	-0.441105	-4.154419
1	6.129438	1.357366	-2.095300
1	4.899501	2.316857	-1.255918
1	6.316146	-0.997183	-1.649600
1	5.218018	-1.761626	-0.488962
44	0.000033	-0.000090	-0.691988

The atomic coordinates (Å) of [*trans*-$Cl_2Rh(PTA)_2]^-$; **3**$_{XRD^-}$.

6	4.817000	14.960500	8.303800
6	7.027200	14.559700	6.643200
6	4.746700	15.804000	5.639900
6	6.825500	16.930300	6.165200
6	4.938200	17.265500	7.566200
6	6.893300	16.208200	8.427900
7	5.427500	16.279700	8.536700
7	7.385600	15.929100	7.074900
7	5.361200	17.026300	6.187100
15	5.202000	14.306100	6.621700
17	5.154000	11.421700	8.204100
45	4.370100	12.239800	6.080700
6	3.923200	9.519100	3.857600
6	1.712900	9.919900	5.518300
6	3.993500	8.675600	6.521600
6	1.914600	7.549300	5.996200
6	3.801900	7.214100	4.595200
6	1.846800	8.271400	3.733600
7	3.312700	8.199900	3.624700
7	1.354600	8.550500	5.086500
7	3.378900	7.453300	5.974300
15	3.538200	10.173500	5.539700
17	3.586100	13.057900	3.957300
1	5.177259	14.245439	9.044465
1	3.733172	15.035364	8.413265
1	7.426935	14.378725	5.643651
1	7.479242	13.833106	7.321142
1	3.660496	15.909611	5.635974
1	5.064291	15.662275	4.606257
1	7.227840	17.904482	6.452301
1	7.150605	16.708177	5.147441
1	5.317010	18.245068	7.866917
1	3.847731	17.289556	7.605846
1	7.262054	15.439364	9.108585
1	7.298658	17.173560	8.738570
1	3.562941	10.234166	3.116940

1	5.007030	9.444237	3.748150
1	1.313227	10.100768	6.517887
1	1.260820	10.646521	4.840416
1	5.079692	8.569917	6.525517
1	3.675862	8.817319	7.555224
1	1.512265	6.575120	5.709083
1	1.589480	7.771416	7.013955
1	3.423042	6.234550	4.294479
1	4.892372	7.189959	4.555586
1	1.478050	9.040209	3.052880
1	1.441486	7.306023	3.422926

The atomic coordinates (Å) of [*trans*-$Cl_2Rh(PTA)_2$]$^-$; **3**$_{opt}^-$.

45	-0.000004	-0.000204	0.999996
15	2.288102	-0.000163	0.938235
6	3.252946	-0.000107	-0.641655
6	3.198920	1.387805	1.763696
6	3.199190	-1.387910	1.763770
7	4.701459	0.000041	-0.414919
7	4.653326	1.225494	1.706451
7	4.653563	-1.225312	1.706510
6	5.128207	-1.185920	0.324507
6	5.127976	1.186122	0.324447
6	5.078929	0.000149	2.380097
1	2.973599	-0.878437	-1.226874
1	2.973421	0.878136	-1.226914
1	2.918327	2.328002	1.283750
1	2.872324	1.438850	2.804188
1	2.872607	-1.438962	2.804265
1	2.918785	-2.328191	1.283880
1	4.780390	-2.078289	-0.198735
1	6.220383	-1.197853	0.341974
1	4.779992	2.078399	-0.198841
1	6.220150	1.198263	0.341911
1	4.691441	0.000135	3.400344
1	6.170447	0.000257	2.423376
15	-2.288102	-0.000188	1.061760
6	-3.198921	1.388056	0.236764
6	-3.199193	-1.387659	0.235766
6	-3.252941	-0.000657	2.641652
7	-4.653327	1.225727	0.293959
7	-4.653565	-1.225079	0.293084
7	-4.701456	-0.000432	2.414922
6	-5.127974	1.185895	1.675951
6	-5.078932	0.000606	-0.380093
6	-5.128207	-1.186147	1.675102
1	-2.918326	2.328092	0.717022
1	-2.872329	1.439448	-0.803713
1	-2.872613	-1.438367	-0.804747

1	-2.918787	-2.328100	0.715343
1	-2.973593	-0.879181	3.226579
1	-2.973414	0.877392	3.227202
1	-4.779988	2.077998	2.199534
1	-6.220147	1.198043	1.658493
1	-4.691447	0.000931	-1.400341
1	-6.170451	0.000728	-0.423370
1	-4.780389	-2.078690	2.198046
1	-6.220383	-1.198074	1.657633
17	-0.170569	0.000103	-1.393555
17	0.170573	-0.000747	3.393545

The atomic coordinates (Å) of $Rh^{+}(PTA)_2$.

45	0.000000	0.000000	1.000000
15	2.382554	0.000000	1.000000
6	3.302010	0.274472	-0.587074
6	3.302010	1.237211	2.031236
6	3.302010	-1.511682	1.555838
7	4.749841	0.240927	-0.393108
7	4.749841	1.086003	1.905203
7	4.749841	-1.326930	1.487905
6	5.193524	-1.053730	0.121695
6	5.193524	1.287500	0.526595
6	5.193524	-0.233769	2.351710
1	3.007050	-0.494676	-1.303285
1	3.006671	1.240013	-1.001865
1	3.007050	2.242041	1.723240
1	3.006671	1.113660	3.074815
1	3.007050	-1.747365	2.580044
1	3.006671	-2.353673	0.927050
1	4.832969	-1.844882	-0.537746
1	6.284667	-1.064269	0.112911
1	4.832969	2.254168	0.171158
1	6.284667	1.300377	0.521861
1	4.832969	-0.409286	3.366588
1	6.284667	-0.236107	2.365229
15	-2.382554	0.000000	1.000000
6	-3.302010	1.394849	0.194683
6	-3.302010	-1.394849	0.194683
6	-3.302010	0.000000	2.610633
7	-4.749841	1.224376	0.293106
7	-4.749841	-1.224376	0.293106
7	-4.749841	0.000000	2.413788
6	-5.193524	1.187993	1.685886
6	-5.193524	-0.000002	-0.371775
6	-5.193524	-1.187991	1.685889
1	-3.007050	2.332495	0.669410
1	-3.006671	1.450944	-0.854685
1	-3.007050	-1.452547	-0.854705

1	-3.006671	-2.331677	0.670788
1	-3.007050	-0.879948	3.185295
1	-3.006671	0.880733	3.183897
1	-4.832969	2.079951	2.200856
1	-6.284667	1.199875	1.692746
1	-4.832969	-0.000004	-1.401719
1	-6.284667	-0.000002	-0.385495
1	-4.832969	-2.079948	2.200862
1	-6.284667	-1.199873	1.692749

The atomic coordinates (Å) of Ni(PTA)$_4$; **4**$_{opt}$.

28	-0.032037	-0.009849	-0.004347
15	-0.676775	2.044957	0.091613
6	-1.435064	2.884522	-1.385043
6	-1.980225	2.587952	1.302628
6	0.522421	3.411362	0.485630
7	-1.817491	4.279416	-1.150657
7	-2.301785	4.015958	1.236940
7	-0.078555	4.747436	0.511158
6	-0.658407	5.090808	-0.785605
6	-2.810000	4.382889	-0.082975
6	-1.126902	4.835894	1.525179
1	-0.721072	2.841880	-2.211954
1	-2.316356	2.315038	-1.692287
1	-2.887632	2.006554	1.118308
1	-1.639838	2.340322	2.311688
1	0.976933	3.203276	1.458001
1	1.326151	3.390841	-0.255304
1	-0.964785	6.138537	-0.750405
1	0.102772	4.978068	-1.560678
1	-3.157989	5.417485	-0.046133
1	-3.660235	3.739373	-0.319259
1	-1.452224	5.875878	1.598951
1	-0.706206	4.535199	2.487177
15	-1.755608	-1.298782	-0.127149
6	-2.261489	-2.076497	-1.739485
6	-1.859069	-2.863558	0.873429
6	-3.452096	-0.680655	0.320323
7	-3.453671	-2.923450	-1.648435
7	-3.096180	-3.622637	0.672751
7	-4.511348	-1.683452	0.181398
6	-4.613067	-2.156973	-1.197426
6	-3.243493	-4.033704	-0.721673
6	-4.267215	-2.833485	1.049106
1	-2.441361	-1.280011	-2.466563
1	-1.424024	-2.670677	-2.114968
1	-1.004359	-3.495381	0.617194

1	-1.761383	-2.605059	1.931267
1	-3.428946	-0.323344	1.353366
1	-3.684639	0.180455	-0.311973
1	-5.499063	-2.791372	-1.270855
1	-4.743753	-1.300659	-1.862625
1	-4.103266	-4.702915	-0.797766
1	-2.348174	-4.584508	-1.018087
1	-5.140437	-3.488428	1.012029
1	-4.145277	-2.475993	2.073886
15	1.134548	-0.479440	1.746563
6	2.831524	0.241656	1.992889
6	0.512713	-0.075890	3.452686
6	1.620089	-2.232313	2.136522
7	3.487207	-0.179054	3.233857
7	1.427287	-0.461147	4.530669
7	2.411026	-2.376809	3.361454
6	3.663521	-1.629048	3.272916
6	2.711285	0.224814	4.404567
6	1.669910	-1.902276	4.527927
1	3.455846	-0.044049	1.142019
1	2.749676	1.331838	1.979160
1	0.325820	0.999960	3.507473
1	-0.448385	-0.576406	3.598155
1	0.710233	-2.832292	2.223547
1	2.187481	-2.630553	1.291030
1	4.268975	-1.882153	4.145960
1	4.203563	-1.937807	2.375217
1	3.308213	0.011310	5.293912
1	2.527762	1.300546	4.360278
1	2.240170	-2.149217	5.426072
1	0.713322	-2.427702	4.569282
15	1.247298	-0.282272	-1.717885
6	0.554509	-0.283794	-3.444276
6	2.637344	0.900452	-2.077496
6	2.261563	-1.827926	-1.924292
7	1.558688	-0.480984	-4.492921
7	3.408978	0.571043	-3.278740

7	3.075153	-1.852716	-3.142641
6	2.239160	-1.764760	-4.338094
6	2.562417	0.580956	-4.469677
6	4.029948	-0.746800	-3.162922
1	-0.198107	-1.073745	-3.514504
1	0.039399	0.666014	-3.611348
1	2.218503	1.904773	-2.183784
1	3.306260	0.921536	-1.212919
1	2.914933	-1.931974	-1.053851
1	1.586241	-2.687765	-1.923278
1	2.878686	-1.926351	-5.208576
1	1.486612	-2.555758	-4.309194
1	3.198668	0.461922	-5.349368
1	2.061983	1.549710	-4.532317
1	4.698140	-0.880125	-4.016494
1	4.624865	-0.784427	-2.247771

The atomic coordinates (Å) of Pd(PTA)$_4$; **5**$_{opt}$.

46	-0.034746	-0.010919	-0.001110
15	-0.734444	2.213239	0.023473
6	-1.420305	3.022185	-1.504776
6	-2.094032	2.780794	1.159143
6	0.445606	3.587615	0.446457
7	-1.812357	4.421508	-1.317689
7	-2.410866	4.207067	1.048815
7	-0.154768	4.923811	0.415697
6	-0.671440	5.240399	-0.914164
6	-2.854862	4.546741	-0.301220
6	-1.250486	5.032921	1.376218
1	-0.667522	2.962677	-2.295397
1	-2.286289	2.446401	-1.842193
1	-2.992007	2.195618	0.943469
1	-1.802668	2.553996	2.188208
1	0.852752	3.399615	1.443548
1	1.283913	3.552002	-0.254456
1	-0.978283	6.288610	-0.915213
1	0.125975	5.111871	-1.649358
1	-3.203358	5.581850	-0.302356
1	-3.693338	3.898365	-0.564784
1	-1.578108	6.074179	1.412939
1	-0.876673	4.752097	2.363286
15	-1.898543	-1.411754	-0.032382
6	-2.359713	-2.364173	-1.562273
6	-2.035641	-2.855506	1.132576
6	-3.605456	-0.748161	0.293872
7	-3.556510	-3.196055	-1.412411
7	-3.268620	-3.632532	0.981569
7	-4.663171	-1.760464	0.236503
6	-4.726235	-2.384483	-1.083471
6	-3.376616	-4.196240	-0.362185
6	-4.447737	-2.806745	1.233493
1	-2.516036	-1.653605	-2.378337
1	-1.513494	-2.996183	-1.844733
1	-1.175759	-3.511651	0.973257

1	-1.967937	-2.480896	2.157461
1	-3.611241	-0.278137	1.280963
1	-3.816986	0.037158	-0.436767
1	-5.611624	-3.023380	-1.111702
1	-4.835019	-1.607515	-1.843331
1	-4.235818	-4.870022	-0.388424
1	-2.474682	-4.776361	-0.569254
1	-5.321475	-3.462010	1.244031
1	-4.354531	-2.337405	2.215294
15	1.278350	-0.433459	1.878861
6	3.053848	0.116281	1.954628
6	0.806897	0.233126	3.550309
6	1.598370	-2.172391	2.456830
7	3.735073	-0.228094	3.205287
7	1.738984	-0.124294	4.622818
7	2.442093	-2.261245	3.651420
6	3.759022	-1.674260	3.412978
6	3.078381	0.393808	4.353200
6	1.827157	-1.574011	4.784852
1	3.591879	-0.334756	1.116539
1	3.087120	1.199673	1.811584
1	0.738533	1.322141	3.481908
1	-0.191178	-0.135957	3.801171
1	0.637102	-2.652178	2.659491
1	2.068318	-2.727461	1.640525
1	4.385935	-1.890422	4.280822
1	4.208161	-2.144060	2.535218
1	3.702005	0.219579	5.232767
1	3.007361	1.470860	4.186423
1	2.421307	-1.777219	5.678493
1	0.824576	-1.980317	4.935341
15	1.307031	-0.382985	-1.871473
6	0.560397	-0.615277	-3.559348
6	2.581994	0.862874	-2.403122
6	2.438237	-1.856729	-1.965248
7	1.542417	-0.838535	-4.623587
7	3.338311	0.474586	-3.596448

7	3.210604	-1.941385	-3.207461
6	2.331385	-2.042095	-4.370449
6	2.455156	0.296118	-4.746792
6	4.069547	-0.771419	-3.376283
1	-0.126231	-1.465162	-3.520092
1	-0.036641	0.268872	-3.798367
1	2.078331	1.814876	-2.591053
1	3.274898	1.024762	-1.573073
1	3.126191	-1.819553	-1.116342
1	1.836710	-2.762551	-1.851413
1	2.953146	-2.240666	-5.246168
1	1.648128	-2.882950	-4.232527
1	3.069837	0.138551	-5.635747
1	1.874563	1.210917	-4.884467
1	4.718083	-0.937974	-4.239163
1	4.695296	-0.668422	-2.487052

The atomic coordinates (Å) of $Pt(PTA)_4$; **6**$_{opt}$.

78	0.097411	-0.012282	-0.007690
15	-0.627307	2.100166	-0.586082
6	-0.558158	3.491770	0.639299
6	0.126325	3.038646	-1.998476
6	-2.394198	2.360989	-1.089395
7	-1.086674	4.756153	0.122104
7	-0.479780	4.354096	-2.217766
7	-2.714650	3.753285	-1.411311
6	-2.491294	4.626063	-0.260434
6	-0.328136	5.209011	-1.042155
6	-1.903394	4.237288	-2.526533
1	-1.119859	3.198268	1.530154
1	0.482379	3.627856	0.945640
1	1.194977	3.158053	-1.803145
1	0.028922	2.434672	-2.904412
1	-2.604230	1.728828	-1.956282
1	-3.038212	2.015187	-0.276617
1	-2.866383	5.620674	-0.512626
1	-3.059507	4.246710	0.590947
1	-0.677618	6.210192	-1.304879
1	0.729258	5.269190	-0.778092
1	-2.270567	5.227201	-2.807210
1	-2.030352	3.566193	-3.378027
15	2.386474	-0.148780	-0.079524
6	3.371707	-0.333871	1.482984
6	3.234853	-1.511012	-1.012947
6	3.397725	1.237428	-0.788359
7	4.818261	-0.402604	1.263434
7	4.696947	-1.445311	-0.950986
7	4.841485	0.992847	-0.750928
6	5.312948	0.813162	0.620578
6	5.172739	-1.547389	0.427158
6	5.195782	-0.196437	-1.522951
1	3.142218	0.512853	2.135279
1	3.036011	-1.238797	1.996907
1	2.895971	-2.468364	-0.608382

1	2.910527	-1.467148	-2.056314
1	3.082817	1.398591	-1.822708
1	3.166757	2.150788	-0.233218
1	6.404156	0.764198	0.599120
1	5.016475	1.678289	1.216480
1	6.262294	-1.624393	0.403791
1	4.770658	-2.456772	0.877544
1	6.285655	-0.257336	-1.568778
1	4.811532	-0.090198	-2.539069
15	-0.893539	-1.508488	-1.456622
6	-2.661617	-2.006873	-1.194600
6	-1.062235	-1.106287	-3.260199
6	-0.231637	-3.225907	-1.694520
7	-3.163187	-2.945808	-2.200370
7	-1.744645	-2.146915	-4.032598
7	-1.008884	-4.026934	-2.643377
6	-2.390841	-4.186631	-2.195681
6	-3.103412	-2.365754	-3.540581
6	-1.018221	-3.413623	-3.969913
1	-2.751772	-2.454340	-0.201102
1	-3.277155	-1.103479	-1.197151
1	-1.607970	-0.163810	-3.355950
1	-0.062386	-0.941951	-3.670404
1	0.802512	-3.153395	-2.040615
1	-0.212898	-3.725428	-0.722369
1	-2.883780	-4.894246	-2.866375
1	-2.393765	-4.605104	-1.187506
1	-3.605574	-3.050356	-4.228102
1	-3.641115	-1.415811	-3.542656
1	-1.495476	-4.111574	-4.661730
1	0.010875	-3.251722	-4.295772
15	-0.702378	-0.462436	2.108764
6	0.134499	0.188708	3.631635
6	-2.416886	0.072429	2.574712
6	-0.867274	-2.212302	2.703106
7	-0.535631	-0.193854	4.876989
7	-2.797751	-0.297455	3.939609

7	-1.423831	-2.324059	4.053252
6	-0.575636	-1.646595	5.031821
6	-1.904700	0.315481	4.921003
6	-2.764946	-1.747256	4.122312
1	1.163747	-0.178670	3.644065
1	0.181131	1.278541	3.560381
1	-2.484886	1.157449	2.458348
1	-3.119940	-0.371304	1.864910
1	-1.501408	-2.759084	2.000110
1	0.120849	-2.678971	2.674456
1	-0.964234	-1.869788	6.028162
1	0.439240	-2.041751	4.959174
1	-2.307927	0.115333	5.916417
1	-1.889363	1.395594	4.764087
1	-3.180027	-1.973343	5.107256
1	-3.394931	-2.216773	3.364499

The atomic coordinates (Å) of $Cl_2Ni(PTA)_2$; *cis*-**7**$_{opt}$.

28	0.782580	3.134143	9.661214
15	0.404226	1.669686	11.253501
15	1.817960	1.709630	8.329828
17	-0.015378	4.735057	10.982260
17	0.802200	4.488285	7.880560
7	1.255781	-0.123818	13.136382
7	-0.791576	-0.704710	11.921080
7	-0.831458	1.043539	13.624964
7	3.169068	-0.596811	7.683393
7	3.789076	1.409650	6.446431
7	1.651598	0.304230	5.990674
6	1.780518	0.841336	12.175133
6	2.773115	1.072150	5.449632
6	-0.584896	0.176167	10.776453
6	-0.608893	2.169026	12.723580
6	0.475103	-1.173920	12.482662
6	0.424926	0.518871	14.152611
6	2.588302	0.092105	8.831461
6	3.293574	2.373669	7.421779
6	0.829752	1.108865	6.888422
6	4.250428	0.180181	7.082977
6	-1.559162	-0.042311	12.974154
6	2.170179	-0.888851	6.656564
1	-2.486707	0.346091	12.551872
1	-1.806911	-0.787305	13.732096
1	3.375693	0.292096	9.560089
1	1.854884	-0.561324	9.303058
1	3.000961	3.296609	6.925729
1	4.075501	2.608234	8.145888
1	-0.017083	0.521348	7.247108
1	0.436169	1.979286	6.363141
1	4.730064	-0.439136	6.323245
1	4.987636	0.424335	7.848989
1	1.342196	-1.440036	7.104520
1	2.640237	-1.521977	5.902368
1	3.249927	0.471687	4.673090

1	2.394233	1.989375	4.997345
1	2.453342	0.336108	11.485154
1	2.358890	1.608071	12.693309
1	-1.547395	0.514312	10.388610
1	-0.093760	-0.374958	9.974904
1	-0.089974	2.971023	13.246719
1	-1.561261	2.567374	12.373845
1	0.249794	-1.938721	13.227493
1	1.074205	-1.629646	11.692803
1	0.189144	-0.224480	14.916017
1	0.987770	1.328835	14.618175

The atomic coordinates (Å) of $Cl_2Ni(PTA)_2$; *trans*-**7**$_{opt}$.

28	0.000000	0.000000	0.000000
15	-0.774447	2.018835	-0.554416
17	1.062316	0.788331	1.747684
7	-0.994158	3.967448	-2.467946
7	-2.968992	3.576248	-1.072189
7	-1.013996	4.715011	-0.136337
6	2.609373	-2.228705	0.634866
6	0.376073	-3.527430	-0.432968
6	2.451730	-3.864113	2.409349
6	0.555654	-4.972476	1.500031
6	2.470564	-4.593120	0.146855
6	-2.609373	2.228705	-0.634866
6	-0.376073	3.527430	0.432968
6	-2.451730	3.864113	-2.409349
6	-0.555654	4.972476	-1.500031
6	-2.470564	4.593120	-0.146855
6	-0.353230	2.675586	-2.230110
15	0.774447	-2.018835	0.554416
17	-1.062316	-0.788331	-1.747684
7	0.994158	-3.967448	2.467946
7	2.968992	-3.576248	1.072189
7	1.013996	-4.715011	0.136337
6	0.353230	-2.675586	2.230110
1	-0.730136	-2.779972	2.310116
1	0.676755	-1.952596	2.979935
1	3.015091	-1.488831	1.326005
1	3.033652	-2.034220	-0.351707
1	0.730136	2.779972	-2.310116
1	-0.676755	1.952596	-2.979935
1	-3.015091	1.488831	-1.326005
1	-3.033652	2.034220	0.351707
1	-0.711952	3.380254	1.460453
1	0.706310	3.661811	0.459037
1	-2.870319	4.818343	-2.734571
1	-2.784207	3.086099	-3.098017
1	0.533852	5.027668	-1.508996

1	-0.955190	5.938775	-1.813479
1	-2.887434	5.556100	-0.447695
1	-2.819482	4.363254	0.860843
1	2.887434	-5.556100	0.447695
1	2.819482	-4.363254	-0.860843
1	-0.533852	-5.027668	1.508996
1	0.955190	-5.938775	1.813479
1	2.870319	-4.818343	2.734571
1	2.784207	-3.086099	3.098017
1	0.711952	-3.380254	-1.460453
1	-0.706310	-3.661811	-0.459037

The atomic coordinates (Å) of $Cl_2Pd(PTA)_2$; *cis*-**8**$_{XRD}$.

46	-0.861100	-4.263700	9.873200
17	-2.344400	-5.376100	11.313100
17	-2.720000	-3.539300	8.588700
15	0.509500	-3.255100	8.393500
15	0.823800	-4.928100	11.194300
7	2.663000	-1.711900	7.681900
7	2.026300	-3.513000	6.119800
7	0.640200	-1.478800	6.286800
7	2.823200	-4.299900	12.934300
7	1.608000	-6.387000	13.371900
7	3.177400	-6.277100	11.487400
6	2.002500	-2.277300	8.860200
6	1.262500	-4.322200	7.083200
6	-0.284700	-2.003200	7.288500
6	3.105200	-2.768500	6.781900
6	1.165700	-2.548600	5.438600
6	1.781200	-0.820500	6.945000
6	1.784000	-3.665900	12.136000
6	0.393500	-6.049100	12.606200
6	2.197600	-5.918000	10.458800
6	2.247200	-5.201000	13.936100
6	2.585700	-7.082500	12.544800
6	3.761100	-5.079600	12.103400
1	1.702753	-1.476420	9.538383
1	2.717771	-2.909613	9.387611
1	1.914092	-5.071013	7.534678
1	0.461012	-4.852753	6.567293
1	-1.132779	-2.478411	6.801119
1	-0.675581	-1.190680	7.902358
1	3.736365	-3.468628	7.331111
1	3.711732	-2.308315	5.999867
1	0.330275	-3.079713	4.979641
1	1.752531	-2.083907	4.644077
1	2.374638	-0.332065	6.169993
1	1.401129	-0.052057	7.619408
1	2.230005	-2.939987	11.458202

1	1.092506	-3.122432	12.782015
1	-0.327391	-5.547547	13.248310
1	-0.070000	-6.955058	12.219831
1	1.777651	-6.816990	10.005264
1	2.695565	-5.351256	9.671038
1	3.054045	-5.533375	14.591148
1	1.521037	-4.650047	14.535327
1	3.393712	-7.422477	13.195289
1	2.115273	-7.961643	12.101040
1	4.167890	-4.433721	11.324147
1	4.580856	-5.400546	12.746966

The atomic coordinates (Å) of $Cl_2Pd(PTA)_2$; *cis*-**8**$_{opt}$.

46	-0.760614	-4.613079	9.655231
17	-2.295044	-5.897007	10.952089
17	-2.418098	-4.309635	7.951949
15	0.564268	-3.384579	8.285747
15	0.851287	-5.020671	11.188302
7	2.817524	-2.025550	7.535781
7	1.702386	-3.346354	5.806849
7	0.707781	-1.249174	6.590042
7	2.703740	-4.158313	12.997200
7	1.579206	-6.261906	13.514954
7	3.257275	-6.187391	11.741210
6	2.244014	-2.710365	8.690018
6	0.978388	-4.240646	6.704458
6	-0.163652	-1.823068	7.609790
6	2.972861	-2.917179	6.387224
6	0.910087	-2.164169	5.466686
6	2.009066	-0.875683	7.135680
6	1.696801	-3.637708	12.077606
6	0.414202	-6.051238	12.662330
6	2.331617	-5.979286	10.631958
6	2.109343	-5.002917	14.031362
6	2.645509	-6.971851	12.813061
6	3.736330	-4.921827	12.296751
1	2.153810	-2.002239	9.514654
1	2.913352	-3.512293	9.001785
1	1.581283	-5.123652	6.923152
1	0.047582	-4.571701	6.243072
1	-1.139982	-2.056045	7.189060
1	-0.303816	-1.113603	8.426902
1	3.546920	-3.795117	6.686105
1	3.533738	-2.380653	5.620443
1	-0.059865	-2.481213	5.082205
1	1.436212	-1.620801	4.680198
1	2.557535	-0.328577	6.367261
1	1.865611	-0.218595	7.994279
1	2.170352	-2.963260	11.367059

1	0.946557	-3.064810	12.624727
1	-0.377910	-5.551379	13.219345
1	0.019159	-7.005827	12.316784
1	1.992695	-6.940127	10.241193
1	2.839793	-5.461755	9.818438
1	2.883579	-5.235258	14.764386
1	1.311341	-4.452507	14.531084
1	3.423315	-7.217867	13.537696
1	2.250083	-7.899837	12.398531
1	4.156872	-4.311086	11.496532
1	4.529991	-5.147256	13.010547

The atomic coordinates (Å) of $Cl_2Pd(PTA)_2$; *trans*-**8**$_{opt}$.

46	0.000000	0.000000	0.000000
15	-0.805445	2.092065	-0.583768
17	1.048818	0.826645	1.910117
7	-1.026240	4.037587	-2.491393
7	-2.997510	3.645348	-1.091147
7	-1.037760	4.779164	-0.157410
6	2.639018	-2.295899	0.658560
6	0.398260	-3.591896	-0.409790
6	2.483868	-3.936316	2.429323
6	0.582624	-5.039309	1.522300
6	2.494877	-4.658550	0.163675
6	-2.639018	2.295899	-0.658560
6	-0.398260	3.591896	0.409790
6	-2.483868	3.936316	-2.429323
6	-0.582624	5.039309	-1.522300
6	-2.494877	4.658550	-0.163675
6	-0.386129	2.744219	-2.259460
15	0.805445	-2.092065	0.583768
17	-1.048818	-0.826645	-1.910117
7	1.026240	-4.037587	2.491393
7	2.997510	-3.645348	1.091147
7	1.037760	-4.779164	0.157410
6	0.386129	-2.744219	2.259460
1	-0.697177	-2.844901	2.342757
1	0.714518	-2.021099	3.007234
1	3.044500	-1.557742	1.352113
1	3.060945	-2.096687	-0.327906
1	0.697177	2.844901	-2.342757
1	-0.714518	2.021099	-3.007234
1	-3.044500	1.557742	-1.352113
1	-3.060945	2.096687	0.327906
1	-0.731041	3.441159	1.437657
1	0.684527	3.723635	0.431209
1	-2.901869	4.892157	-2.750094
1	-2.819782	3.161037	-3.119283
1	0.506854	5.092794	-1.534276

1	-0.981555	6.007027	-1.831655
1	-2.911273	5.622885	-0.460327
1	-2.840957	4.426156	0.844330
1	2.911273	-5.622885	0.460327
1	2.840957	-4.426156	-0.844330
1	-0.506854	-5.092794	1.534276
1	0.981555	-6.007027	1.831655
1	2.901869	-4.892157	2.750094
1	2.819782	-3.161037	3.119283
1	0.731041	-3.441159	-1.437657
1	-0.684527	-3.723635	-0.431209

The atomic coordinates (Å) of $Cl_2Pt(PTA)_2$; *cis*-**9**$_{XRD}$.

78	1.070500	3.201800	9.828400
15	0.454600	1.631900	11.278300
15	1.968300	1.755800	8.390200
17	0.068600	4.826600	11.197400
17	1.710200	4.974100	8.388900
7	1.208200	-0.249800	13.107300
7	-0.858300	-0.704500	11.818700
7	-0.858700	0.998400	13.602300
7	3.285900	-0.537900	7.666000
7	3.630200	1.427200	6.231000
7	1.483900	0.222100	6.173200
6	1.784900	0.732700	12.184700
6	2.482500	0.994600	5.433600
6	-0.574400	0.212300	10.709300
6	-0.570400	2.138700	12.722100
6	0.385200	-1.240300	12.394600
6	0.367500	0.403600	14.112800
6	2.840400	0.205900	8.858300
6	3.227800	2.423500	7.226800
6	0.791800	1.071400	7.159400
6	4.227500	0.247200	6.862800
6	-1.610700	-0.026000	12.885800
6	2.148900	-0.904500	6.835800
1	-2.507789	0.420267	12.455586
1	-1.915911	-0.787031	13.606166
1	3.701896	0.453774	9.480694
1	2.183602	-0.441267	9.440616
1	2.807605	3.293504	6.728697
1	4.096285	2.758787	7.796277
1	0.014645	0.493954	7.662845
1	0.300815	1.903845	6.652581
1	4.612856	-0.400849	6.074164
1	5.059918	0.561016	7.493422
1	1.419039	-1.448610	7.436335
1	2.511140	-1.576807	6.055268
1	2.856988	0.365236	4.623955

1	2.001597	1.869502	4.994529
1	2.457515	0.233547	11.489855
1	2.375240	1.463630	12.739808
1	-1.505758	0.601871	10.295124
1	-0.066443	-0.332889	9.912995
1	-0.038119	2.911147	13.275251
1	-1.499897	2.577792	12.360621
1	0.110865	-2.017367	13.109316
1	0.979184	-1.696344	11.601512
1	0.090387	-0.351191	14.851355
1	0.954520	1.173054	14.616609

The atomic coordinates (Å) of $Cl_2Pt(PTA)_2$; *cis*-**9**$_{opt}$.

78	0.684166	3.083503	9.615349
15	0.330561	1.606700	11.264075
15	1.816403	1.686199	8.267121
17	-0.525301	4.750743	10.847078
17	0.949065	4.687313	7.835325
7	1.290898	-0.104053	13.160895
7	-0.801712	-0.745963	12.059573
7	-0.783962	1.063679	13.701225
7	3.107790	-0.630944	7.608854
7	3.862689	1.396824	6.482464
7	1.717290	0.383440	5.871480
6	1.758511	0.829094	12.139995
6	2.891206	1.131858	5.421297
6	-0.654272	0.093682	10.874015
6	-0.620052	2.159353	12.750668
6	0.492392	-1.184402	12.582312
6	0.501243	0.570678	14.189124
6	2.487932	0.030587	8.753217
6	3.348294	2.343545	7.467283
6	0.879764	1.181583	6.761655
6	4.246120	0.130434	7.098384
6	-1.528313	-0.051639	13.121827
6	2.158680	-0.850317	6.518102
1	-2.477834	0.313235	12.728500
1	-1.733951	-0.770897	13.916227
1	3.236779	0.165769	9.534653
1	1.694077	-0.603189	9.148566
1	3.107402	3.292503	6.991692
1	4.098879	2.530135	8.237203
1	0.000082	0.610626	7.064220
1	0.537526	2.084411	6.254171
1	4.747893	-0.478079	6.344334
1	4.946411	0.322158	7.912272
1	1.289233	-1.388060	6.898442
1	2.649596	-1.471570	5.767461
1	3.388984	0.544718	4.647888

1	2.567516	2.077956	4.986117
1	2.395331	0.301820	11.432375
1	2.358272	1.618428	12.596132
1	-1.635102	0.405763	10.511285
1	-0.181372	-0.474771	10.073246
1	-0.092991	2.990542	13.218718
1	-1.593474	2.528289	12.428942
1	0.306054	-1.921078	13.365175
1	1.060542	-1.666049	11.785149
1	0.308621	-0.144105	14.990871
1	1.076716	1.402915	14.596323

The atomic coordinates (Å) of $Cl_2Pt(PTA)_2$; *trans*-**9**$_{XRD}$.

78	0.000000	0.000000	0.000000
15	-0.809700	2.029400	-0.565900
17	0.905300	0.798100	2.010400
7	-1.005200	3.980900	-2.505500
7	-2.991700	3.613200	-1.078500
7	-1.010300	4.735400	-0.147800
6	2.626200	-2.251600	0.633200
6	0.390000	-3.531000	-0.415700
6	2.468300	-3.893800	2.415200
6	0.556600	-4.979700	1.512400
6	2.470700	-4.619300	0.141200
6	-2.626200	2.251600	-0.633200
6	-0.390000	3.531000	0.415700
6	-2.468300	3.893800	-2.415200
6	-0.556600	4.979700	-1.512400
6	-2.470700	4.619300	-0.141200
6	-0.388800	2.670000	-2.227400
15	0.809700	-2.029400	0.565900
17	-0.905300	-0.798100	-2.010400
7	1.005200	-3.980900	2.505500
7	2.991700	-3.613200	1.078500
7	1.010300	-4.735400	0.147800
6	0.388800	-2.670000	2.227400
1	-0.696601	-2.755487	2.307218
1	0.715577	-1.944688	2.973748
1	3.046856	-1.514859	1.320031
1	3.050525	-2.064888	-0.354528
1	0.696601	2.755487	-2.307218
1	-0.715577	1.944688	-2.973748
1	-3.046856	1.514859	-1.320031
1	-3.050525	2.064888	0.354528
1	-0.721572	3.389960	1.445295
1	0.694893	3.647540	0.437435
1	-2.877252	4.853135	-2.738198
1	-2.819357	3.121342	-3.101430
1	0.533429	5.021179	-1.524063

1	-0.943439	5.950700	-1.827345
1	-2.886290	5.587433	-0.427368
1	-2.808571	4.378910	0.867092
1	2.886290	-5.587433	0.427368
1	2.808571	-4.378910	-0.867092
1	-0.533429	-5.021179	1.524063
1	0.943439	-5.950700	1.827345
1	2.877252	-4.853135	2.738198
1	2.819357	-3.121342	3.101430
1	0.721572	-3.389960	-1.445295
1	-0.694893	-3.647540	-0.437435

The atomic coordinates (Å) of $Cl_2Pt(PTA)_2$; *trans*-**9**$_{opt}$.

78	0.000000	0.000000	0.000000
15	-0.804599	2.087559	-0.589747
17	1.029524	0.837754	1.926806
7	-1.026465	4.035075	-2.491997
7	-2.996358	3.641366	-1.089768
7	-1.033733	4.770853	-0.155891
6	2.637094	-2.291091	0.661006
6	0.394141	-3.581971	-0.407774
6	2.484088	-3.935122	2.427952
6	0.580496	-5.033778	1.521038
6	2.491081	-4.651540	0.160384
6	-2.637094	2.291091	-0.661006
6	-0.394141	3.581971	0.407774
6	-2.484088	3.935122	-2.427952
6	-0.580496	5.033778	-1.521038
6	-2.491081	4.651540	-0.160384
6	-0.386905	2.740746	-2.263905
15	0.804599	-2.087559	0.589747
17	-1.029524	-0.837754	-1.926806
7	1.026465	-4.035075	2.491997
7	2.996358	-3.641366	1.089768
7	1.033733	-4.770853	0.155891
6	0.386905	-2.740746	2.263905
1	-0.696477	-2.839902	2.348050
1	0.716612	-2.018978	3.012540
1	3.043929	-1.553893	1.355045
1	3.056241	-2.088714	-0.326047
1	0.696477	2.839902	-2.348050
1	-0.716612	2.018978	-3.012540
1	-3.043929	1.553893	-1.355045
1	-3.056241	2.088714	0.326047
1	-0.725920	3.428319	1.435543
1	0.688810	3.712715	0.428529
1	-2.901568	4.892180	-2.745689
1	-2.821853	3.161811	-3.119213
1	0.509017	5.086251	-1.534362

1	-0.979028	6.002691	-1.827096
1	-2.906871	5.617124	-0.453727
1	-2.835743	4.416657	0.847549
1	2.906871	-5.617124	0.453727
1	2.835743	-4.416657	-0.847549
1	-0.509017	-5.086251	1.534362
1	0.979028	-6.002691	1.827096
1	2.901568	-4.892180	2.745689
1	2.821853	-3.161811	3.119213
1	0.725920	-3.428319	-1.435543
1	-0.688810	-3.712715	-0.428529

The atomic coordinates (Å) of $Cu^{+}(PTA)_4$; **10**$_{opt}^{+}$.

29	-0.029244	0.009391	-0.002901
15	0.763730	0.014062	2.149195
6	0.871134	1.611648	3.081925
6	-0.090976	-0.971226	3.467469
6	2.485008	-0.579509	2.496884
7	1.405798	1.434044	4.430831
7	0.559306	-0.839893	4.770020
7	2.828191	-0.493331	3.915199
6	2.760670	0.884193	4.400317
6	0.559772	0.548611	5.229312
6	1.939490	-1.319900	4.730755
1	1.506898	2.303541	2.525251
1	-0.125702	2.054978	3.135069
1	-1.128056	-0.638699	3.543599
1	-0.107792	-2.021413	3.168546
1	2.575066	-1.615138	2.162316
1	3.189494	0.014750	1.911155
1	3.155338	0.902749	5.418092
1	3.390303	1.516516	3.772262
1	0.929766	0.563950	6.256323
1	-0.463321	0.928162	5.223167
1	2.324917	-1.323373	5.752231
1	1.952735	-2.342910	4.351284
15	-2.296182	0.360135	-0.005066
6	-2.979388	2.017257	-0.478493
6	-3.390206	-0.662795	-1.098657
6	-3.268288	0.147834	1.559638
7	-4.439423	2.056208	-0.423556
7	-4.801039	-0.303859	-0.969726
7	-4.693848	0.408969	1.371392
6	-4.931665	1.780942	0.925648
6	-5.035756	1.089922	-1.344529
6	-5.282009	-0.507968	0.395760
1	-2.567361	2.772000	0.195010
1	-2.644481	2.260429	-1.489643
1	-3.071751	-0.533147	-2.135507

1	-3.257967	-1.716776	-0.843043
1	-3.127039	-0.871619	1.925718
1	-2.870114	0.828163	2.316269
1	-6.009169	1.956315	0.933619
1	-4.462445	2.472887	1.626823
1	-6.114340	1.258187	-1.358352
1	-4.644972	1.262470	-2.348504
1	-6.363013	-0.355485	0.398914
1	-5.075760	-1.535615	0.699024
15	0.446429	-2.017661	-0.965609
6	2.197521	-2.445769	-1.399268
6	0.040533	-3.576605	-0.049832
6	-0.316542	-2.492676	-2.587356
7	2.315362	-3.780837	-1.983369
7	0.416019	-4.775584	-0.796937
7	0.101836	-3.821174	-3.031213
6	1.549370	-3.890579	-3.224046
6	1.854651	-4.814172	-1.057237
6	-0.292176	-4.853753	-2.073574
1	2.576915	-1.703608	-2.105201
1	2.812381	-2.383125	-0.498986
1	0.558993	-3.566797	0.911293
1	-1.031317	-3.592736	0.158902
1	-1.403554	-2.467071	-2.487466
1	-0.041740	-1.750143	-3.339341
1	1.783033	-4.854354	-3.680712
1	1.858820	-3.098558	-3.907853
1	2.092631	-5.786914	-1.492093
1	2.392872	-4.715161	-0.113205
1	-0.077435	-5.826763	-2.519730
1	-1.366049	-4.784151	-1.892835
15	1.029461	1.664142	-1.186520
6	0.618894	3.447354	-0.888557
6	2.872803	1.827989	-1.079520
6	0.897125	1.698264	-3.034469
7	1.399251	4.347116	-1.735920
7	3.383510	2.920648	-1.905525

7	1.642811	2.807822	-3.626225
6	1.145270	4.099093	-3.154589
6	2.833086	4.209007	-1.486546
6	3.069721	2.715993	-3.318714
1	-0.445513	3.601826	-1.077003
1	0.800187	3.682029	0.162375
1	3.153019	1.996943	-0.037513
1	3.329874	0.886415	-1.391205
1	1.273163	0.753170	-3.431780
1	-0.156999	1.772457	-3.310764
1	1.643695	4.882860	-3.728331
1	0.072118	4.160425	-3.342462
1	3.349251	4.993513	-2.043392
1	3.027599	4.353090	-0.422561
1	3.589082	3.484844	-3.894101
1	3.441605	1.738544	-3.630274

The atomic coordinates (Å) of [LCS$_2$]Cu(PTA); **11**$_{opt.}$

15	2.296397	-0.011181	0.203220
7	4.718474	1.084145	0.974809
7	4.596865	0.235279	-1.319272
7	4.703204	-1.329391	0.562651
6	3.278215	1.226248	1.177175
1	2.965734	2.233149	0.890999
1	3.041157	1.104974	2.236153
6	3.139813	0.263629	-1.427572
1	2.810013	-0.503396	-2.132327
1	2.821292	1.227425	-1.831431
6	3.260987	-1.513533	0.709695
1	3.023655	-1.746863	1.749720
1	2.937207	-2.364116	0.106120
6	5.085490	1.283407	-0.425155
1	4.704198	2.248938	-0.762322
1	6.175449	1.301220	-0.489880
6	5.069947	-1.056549	-0.825182
1	4.676320	-1.850036	-1.462993
1	6.159688	-1.066073	-0.894643
6	5.190086	-0.232969	1.397636
1	6.281076	-0.232123	1.351740
1	4.887670	-0.407699	2.431383
29	0.105818	-0.008419	0.323693
16	-1.192470	-0.044619	2.307206
16	-4.161475	0.021728	2.555818
7	-1.128844	1.474926	-0.549212
7	-2.449754	1.208915	-0.493049
7	-1.140096	-1.426323	-0.633581
7	-2.460689	-1.187978	-0.506613
6	-1.013023	2.653625	-1.133455
6	-2.285353	3.160813	-1.451528
6	-3.185468	2.214283	-1.026841
6	0.324482	3.256757	-1.387865
6	-4.671658	2.196871	-1.085320
6	-1.032010	-2.610418	-1.209893
6	-2.308690	-3.146889	-1.451229

6	-3.203910	-2.213927	-0.985840
6	0.302783	-3.206145	-1.494413
6	-4.691161	-2.224726	-0.959662
6	-2.953282	0.009644	0.139645
6	-2.751993	-0.003086	1.674535
1	-2.518215	4.099649	-1.924827
1	0.642950	3.078317	-2.416983
1	0.300572	4.334126	-1.227040
1	1.065878	2.820873	-0.718827
1	-5.108218	2.087107	-0.090808
1	-5.026471	3.131329	-1.514389
1	-5.036884	1.378387	-1.709570
1	-2.547699	-4.092552	-1.907567
1	0.620901	-3.852147	-0.673647
1	0.274735	-3.806456	-2.403050
1	1.048108	-2.420192	-1.616120
1	-5.109202	-1.414911	-1.561676
1	-5.052660	-3.167037	-1.365279
1	-5.070173	-2.118734	0.058692
1	-4.024297	0.015746	-0.023728

The atomic coordinates (Å) of ClAu(PTA); **12**$_{XRD}$.

79	0.835400	0.751800	1.643000
15	2.327800	-0.609900	2.578900
17	-0.596300	2.190200	0.552300
7	4.264400	-0.955200	4.478400
7	4.609800	-2.081600	2.286900
7	2.928400	-2.936600	3.901400
6	3.311200	0.048700	3.998000
6	3.701000	-1.228100	1.525000
6	1.793200	-2.176300	3.329100
6	3.593500	-2.193000	4.927100
6	5.204700	-1.343700	3.389100
6	3.916000	-3.284700	2.814500
1	3.843821	0.947166	3.682912
1	2.626008	0.329630	4.799655
1	3.281772	-1.781677	0.683155
1	4.246653	-0.375510	1.117941
1	1.287599	-2.786554	2.579964
1	1.068634	-1.962384	4.117475
1	4.360385	-2.828118	5.374659
1	2.874924	-1.931265	5.707160
1	5.975638	-1.969894	3.841193
1	5.679031	-0.441096	2.999725
1	4.663165	-3.949348	3.246391
1	3.392997	-3.790910	2.005502

The atomic coordinates (Å) of ClAu(PTA); **12**opt.

79	0.818512	0.765340	1.625343
15	2.349430	-0.589156	2.565192
17	-0.765064	2.164957	0.652420
7	4.276549	-0.974458	4.456903
7	4.613086	-2.088570	2.302309
7	2.917481	-2.921902	3.860024
6	3.342623	0.045214	3.986136
6	3.727929	-1.230912	1.518568
6	1.786285	-2.184966	3.302578
6	3.579271	-2.171279	4.926932
6	5.226059	-1.361519	3.413735
6	3.906147	-3.253341	2.834360
1	3.887503	0.936563	3.671342
1	2.666515	0.335816	4.791793
1	3.308913	-1.790103	0.680539
1	4.287165	-0.388008	1.109719
1	1.293658	-2.779981	2.532079
1	1.052274	-1.977626	4.082752
1	4.314467	-2.828229	5.394750
1	2.841332	-1.886364	5.677888
1	5.977797	-2.010120	3.866512
1	5.722871	-0.469629	3.029660
1	4.645332	-3.919925	3.281774
1	3.413590	-3.780160	2.016123

The atomic coordinates (Å) of IAu(PTA); **14**$_{opt}$.

79	0.811224	0.772380	1.620838
15	2.357474	-0.597235	2.570417
53	-0.963782	2.345319	0.529993
7	4.285508	-0.982507	4.462791
7	4.622629	-2.096648	2.308530
7	2.927237	-2.930646	3.866006
6	3.351041	0.036440	3.992139
6	3.737501	-1.239299	1.524709
6	1.796105	-2.194465	3.307852
6	3.588812	-2.179719	4.932709
6	5.235264	-1.369234	3.419754
6	3.915933	-3.261694	2.840274
1	3.895399	0.927876	3.677039
1	2.674865	0.326523	4.797877
1	3.318716	-1.798894	0.686965
1	4.296726	-0.396278	1.116050
1	1.305239	-2.789633	2.536323
1	1.061235	-1.988093	4.087417
1	4.324720	-2.836243	5.399944
1	2.850971	-1.895351	5.683970
1	5.987606	-2.017079	3.872685
1	5.731512	-0.477028	3.035681
1	4.654861	-3.928692	3.287470
1	3.422927	-3.788157	2.021997

The atomic coordinates (Å) of Au^{+}(PTA).

79	0.811026	0.717501	1.685459
15	2.341622	-0.610706	2.593366
7	4.274010	-0.970425	4.451926
7	4.595834	-2.075838	2.292797
7	2.924923	-2.925696	3.865218
6	3.331679	0.048383	4.001468
6	3.702639	-1.225559	1.513003
6	1.776779	-2.205343	3.324897
6	3.590890	-2.173150	4.928586
6	5.216866	-1.345602	3.398053
6	3.904206	-3.248948	2.827495
1	3.864420	0.943009	3.677496
1	2.659147	0.330342	4.812302
1	3.274791	-1.784971	0.680430
1	4.248499	-0.374881	1.103717
1	1.283401	-2.798546	2.554154
1	1.049928	-2.001799	4.111984
1	4.336477	-2.824803	5.386012
1	2.859189	-1.896134	5.688074
1	5.978644	-1.988616	3.840782
1	5.701376	-0.448624	3.011121
1	4.652656	-3.910758	3.265251
1	3.407024	-3.777396	2.013542

The atomic coordinates (Å) of $F_5C_6Au(PTA)$; **15**XRD.

79	8.209100	5.607900	12.796600
9	11.333900	5.538500	13.910200
9	12.835900	7.464100	15.029900
9	11.851800	9.985100	15.311700
9	9.306100	10.549400	14.485900
9	7.833400	8.658000	13.296800
6	9.533300	7.030500	13.525900
6	10.793100	6.793600	13.987000
6	11.593900	7.754000	14.591400
6	11.090400	9.020600	14.743600
6	9.823600	9.306400	14.326100
6	9.075400	8.310900	13.717300
15	6.495000	4.206900	12.290200
7	4.654200	2.366600	13.132500
7	5.308300	2.255600	10.761900
7	3.842600	4.083200	11.554600
6	5.827800	3.105700	13.610300
6	6.577400	2.983200	10.909600
6	4.930100	5.055800	11.824400
6	4.972600	1.523900	11.979600
6	4.187700	3.173500	10.453000
6	3.554900	3.275500	12.752700
1	6.604029	2.404566	13.921089
1	5.559071	3.710530	14.477428
1	6.812797	3.508709	9.982880
1	7.385096	2.276462	11.105406
1	4.628629	5.723285	12.632662
1	5.102855	5.664345	10.935793
1	5.803300	0.865317	12.240941
1	4.099146	0.904309	11.768863
1	4.441688	3.759529	9.569011
1	3.310634	2.564383	10.231044
1	2.674060	2.666346	12.545305
1	3.333048	3.939484	13.588811

The atomic coordinates (Å) of $F_5C_6Au(PTA)$; **15**$_{opt}$.

79	7.894469	5.837396	13.118523
9	10.956955	5.448489	14.052610
9	12.840115	7.168040	14.846918
9	12.307562	9.824482	14.967901
9	9.851019	10.743586	14.279330
9	7.949006	9.046028	13.481407
6	9.365214	7.168107	13.730214
6	10.634291	6.753486	14.092223
6	11.632171	7.618385	14.508015
6	11.365810	8.974152	14.571544
6	10.111144	9.437641	14.219175
6	9.147455	8.531807	13.809875
15	6.271545	4.355196	12.452302
7	4.633207	2.250672	13.067768
7	5.478362	2.345344	10.771777
7	3.743254	3.910548	11.503078
6	5.655106	3.104404	13.666181
6	6.621312	3.212775	11.041247
6	4.637974	5.002025	11.877279
6	5.155308	1.504782	11.923800
6	4.291203	3.115993	10.404775
6	3.470700	3.023605	12.633057
1	6.495181	2.497927	14.009089
1	5.247012	3.623122	14.535417
1	6.858610	3.804132	10.155089
1	7.499573	2.610446	11.280058
1	4.189267	5.596828	12.674848
1	4.797638	5.662977	11.023691
1	6.046525	0.954467	12.229176
1	4.394475	0.786037	11.614330
1	4.533684	3.775301	9.570002
1	3.521095	2.414579	10.078880
1	2.692784	2.320734	12.329571
1	3.095828	3.612669	13.471283

The atomic coordinates (Å) of $H_5C_6SAu(PTA)$; **16**$_{XRD}$.

79	5.217700	2.865600	16.106300
16	6.596500	1.537700	17.392500
15	3.960200	4.033400	14.652600
7	3.084300	6.409100	13.579300
7	1.527000	4.507600	13.568300
7	3.420700	4.531900	12.005600
6	3.945600	5.898600	14.623500
6	4.316000	3.747200	12.862500
6	2.133400	3.755700	14.647200
6	1.694900	5.975000	13.748500
6	2.046300	4.177800	12.242100
6	3.559500	5.962800	12.227500
6	5.485900	0.340800	18.089300
6	4.177200	0.650000	18.433100
6	3.352500	-0.288600	19.006200
6	3.823900	-1.592300	19.240900
6	5.150900	-1.894200	18.902500
6	5.954100	-0.959200	18.327600
1	4.967347	6.253713	14.477420
1	3.610883	6.257109	15.599305
1	4.202317	2.685129	12.638477
1	5.354608	4.017085	12.664583
1	1.716347	4.064373	15.607141
1	1.933775	2.689068	14.524680
1	1.082180	6.476943	12.999605
1	1.344239	6.257822	14.741945
1	1.425773	4.699733	11.511032
1	1.921241	3.104427	12.077709
1	4.603164	6.257438	12.115809
1	2.962118	6.487802	11.481645
1	3.807027	1.654997	18.271514
1	2.335307	-0.026352	19.270317
1	3.183395	-2.339747	19.689589
1	5.532035	-2.892246	19.084776
1	6.972928	-1.207049	18.055352

The atomic coordinates (Å) of $H_5C_6SAu(PTA)$; **16**$_{opt}$.

79	5.191316	2.720454	15.934450
16	6.509502	1.456936	17.381195
15	3.958586	3.977350	14.485863
7	3.151807	6.437831	13.605139
7	1.487981	4.643498	13.519805
7	3.337262	4.675725	11.915411
6	4.025378	5.820971	14.599159
6	4.237128	3.805373	12.666844
6	2.121994	3.769326	14.501802
6	1.754794	6.053451	13.800630
6	1.934980	4.342568	12.160374
6	3.549664	6.084721	12.243106
6	5.452991	0.258791	18.150427
6	4.081490	0.436358	18.339611
6	3.321682	-0.533813	18.974482
6	3.912469	-1.697582	19.445105
6	5.276737	-1.879235	19.270164
6	6.038280	-0.915295	18.628009
1	5.054929	6.150411	14.448495
1	3.725121	6.126728	15.602921
1	4.078158	2.764697	12.379078
1	5.275139	4.054749	12.440285
1	1.746995	3.995572	15.501585
1	1.879995	2.726960	14.287009
1	1.142352	6.653505	13.125351
1	1.459478	6.278370	14.826440
1	1.324121	4.924808	11.468306
1	1.775386	3.283035	11.955046
1	4.602123	6.333313	12.099464
1	2.955360	6.684828	11.551820
1	3.606118	1.344644	17.990530
1	2.258341	-0.373421	19.105698
1	3.317343	-2.452586	19.942554
1	5.755458	-2.781447	19.631109
1	7.100823	-1.072444	18.489136

The atomic coordinates (Å) of [(py[b]-H)ClAu]+(PTA); **17**XRD.

6	4.955200	12.345200	4.922500
6	4.901700	13.483600	4.133800
6	5.844000	14.499500	4.322700
6	6.819500	14.361500	5.289900
6	6.855900	13.229300	6.088500
6	5.926500	12.203200	5.918400
6	5.916500	11.005100	6.844000
6	4.628200	10.968800	7.620000
6	4.563100	11.038800	8.999100
6	3.341400	11.058300	9.635500
6	2.185000	10.985600	8.893300
6	2.286300	10.908400	7.526800
6	3.442100	9.059100	1.847500
6	2.636200	11.736600	1.480700
6	5.398200	11.122400	1.763900
6	4.986800	9.479700	0.017800
6	2.660800	9.987300	-0.242000
6	4.294100	11.738800	-0.311900
7	3.474500	10.911600	6.902600
7	3.621800	9.076900	0.389300
7	5.302900	10.881200	0.318200
7	2.905400	11.417000	0.059800
15	3.770100	10.737500	2.533200
17	1.955900	9.127100	4.770000
79	3.571600	10.848700	4.805900
1	4.382031	11.620369	-1.392173
1	4.499118	12.779546	-0.059718
1	1.651706	9.723771	0.073443
1	2.734548	9.865177	-1.322744
1	5.700454	8.832309	0.526958
1	5.094270	9.337309	-1.057994
1	4.125807	8.345005	2.307877
1	2.425458	8.769100	2.103754
1	5.666840	12.159633	1.958749
1	6.162416	10.484416	2.208807
1	1.604850	11.489940	1.733766

1	2.788071	12.801303	1.654843
1	5.799329	15.389390	3.708313
1	4.131373	13.615310	3.385507
1	7.553241	15.143739	5.436219
1	7.612284	13.135204	6.858576
1	6.020918	10.077069	6.276089
1	6.751883	11.056431	7.538209
1	3.296969	11.125315	10.714568
1	5.483162	11.085110	9.564905
1	1.209130	10.993309	9.356134
1	1.412119	10.843385	6.895739

The atomic coordinates (Å) of [(py^{b}-H)ClAu]$^{+}$(PTA); **17**$_{opt}$.

6	9.874991	12.756961	1.776484
6	9.965531	13.912691	2.535908
6	9.013956	14.912635	2.384214
6	7.980889	14.751404	1.475947
6	7.917606	13.607116	0.695724
6	8.867800	12.601265	0.829898
6	8.867618	11.410593	-0.097777
6	10.130730	11.383212	-0.917876
6	10.106250	11.387724	-2.301607
6	11.294804	11.399400	-3.008947
6	12.494365	11.404088	-2.317391
6	12.459919	11.386521	-0.941367
6	10.862105	9.268520	4.644573
6	12.393667	11.588280	5.193550
6	9.568542	11.753084	5.003887
6	11.953629	9.776893	6.743326
6	10.861922	11.873552	7.048021
6	9.579627	9.917838	6.589433
7	11.304544	11.378255	-0.267258
7	10.765481	9.218898	6.096698
7	12.083503	11.224411	6.570993
7	9.647452	11.367269	6.407742
15	11.011938	11.028190	4.117085
17	13.027909	9.646915	1.917587
79	11.282020	11.286587	1.856307
1	10.776058	14.057462	3.238819
1	9.486414	9.715995	7.656933
1	8.694803	9.530340	6.083950
1	10.779418	11.689603	8.119627
1	10.937060	12.949018	6.884911
1	12.844100	9.284682	6.352023
1	11.883807	9.570994	7.811766
1	9.575235	12.837495	4.911255
1	8.647962	11.382712	4.551274
1	13.316227	11.109666	4.863667
1	12.514179	12.667924	5.103565

1	9.975969	8.829983	4.184615
1	11.737129	8.723317	4.292870
1	13.363764	11.368762	-0.349956
1	13.445716	11.418014	-2.828170
1	11.283934	11.408088	-4.090575
1	9.154817	11.386073	-2.813739
1	8.789526	10.478238	0.468308
1	8.010872	11.450413	-0.766165
1	7.231622	15.523654	1.360818
1	7.127040	13.494973	-0.036442
1	9.086292	15.814218	2.978478

The atomic coordinates (Å) of $Cl_2Hg(PTA)$; **18**$_{opt}$(gas).

15	-0.419676	2.311244	0.149198
6	0.433272	3.321284	-1.155325
6	-2.162949	2.717657	-0.346127
6	-0.265941	3.524479	1.547469
7	-0.084172	4.682989	-1.215375
7	-2.363966	4.152930	-0.504811
7	-0.700886	4.858515	1.147128
6	0.107536	5.386024	0.049905
6	-1.505746	4.702066	-1.551858
6	-2.104168	4.871802	0.739254
1	1.505300	3.344209	-0.947822
1	0.295760	2.822677	-2.117123
1	-2.380946	2.200332	-1.282835
1	-2.850269	2.331523	0.409730
1	-0.865599	3.178223	2.391614
1	0.773133	3.559235	1.880916
1	-0.168051	6.432066	-0.098523
1	1.162180	5.346145	0.328512
1	-1.799744	5.739953	-1.720949
1	-1.664003	4.144017	-2.475928
1	-2.403880	5.912233	0.598333
1	-2.714798	4.444734	1.536894
80	0.109524	-0.351746	-0.139771
17	-0.607383	-0.277390	-2.384448
17	0.942566	-1.353052	1.785763

The atomic coordinates (Å) of $Cl_2Hg(PTA)$; **18**$_{opt}$(water).

15	-0.486588	2.327557	-0.053524
6	0.372840	3.389760	-1.298617
6	-2.232718	2.783716	-0.453263
6	-0.250370	3.392707	1.437122
7	-0.115375	4.764492	-1.224106
7	-2.388903	4.235546	-0.486343
7	-0.659052	4.767515	1.164333
6	0.135094	5.360514	0.088550
6	-1.546731	4.848167	-1.513286
6	-2.074740	4.847082	0.805301
1	1.446953	3.363520	-1.109774
1	0.198404	2.983141	-2.295864
1	-2.500724	2.355597	-1.420381
1	-2.895268	2.352482	0.298378
1	-0.835672	2.983285	2.261986
1	0.800484	3.364716	1.729185
1	-0.115212	6.421023	0.030111
1	1.194505	5.266438	0.330407
1	-1.815025	5.903726	-1.582554
1	-1.748548	4.374848	-2.474896
1	-2.350234	5.901757	0.754087
1	-2.670959	4.370338	1.584307
80	0.145822	-0.141892	0.141491
17	-0.253184	-0.920665	-2.274437
17	0.912292	-1.293221	2.185608

The atomic coordinates (Å) of $I_2Hg(PTA)$; **20**$_{opt}$(gas).

15	-0.437534	2.322543	0.100224
6	0.419591	3.349131	-1.186345
6	-2.180865	2.744506	-0.375746
6	-0.266078	3.497373	1.526568
7	-0.090507	4.714408	-1.212267
7	-2.370295	4.184340	-0.501605
7	-0.694021	4.842250	1.156807
6	0.112808	5.388241	0.067443
6	-1.513624	4.750181	-1.541135
6	-2.099356	4.873900	0.757020
1	1.492221	3.359068	-0.981777
1	0.275903	2.873512	-2.158970
1	-2.409500	2.249137	-1.321894
1	-2.864936	2.345974	0.376385
1	-0.863651	3.134640	2.365130
1	0.775124	3.515650	1.854318
1	-0.155993	6.439069	-0.056763
1	1.168485	5.334710	0.339383
1	-1.801388	5.793299	-1.686248
1	-1.680346	4.213455	-2.476307
1	-2.392903	5.918976	0.640524
1	-2.708788	4.433219	1.548009
80	0.112195	-0.287508	-0.083130
53	-0.618647	-0.582632	-2.646606
53	1.059202	-1.405293	2.120804

The atomic coordinates (Å) of $I_2Hg(PTA)$; **20**$_{opt}$(water).

15	1.709379	-0.457760	-0.034754
6	2.654130	0.989232	-0.683866
6	2.543137	-0.632643	1.604427
6	2.578744	-1.807369	-0.951264
7	4.090854	0.764313	-0.556367
7	3.992299	-0.654687	1.436573
7	4.023498	-1.672074	-0.789637
6	4.521203	-0.404287	-1.324115
6	4.488470	0.585151	0.840248
6	4.425995	-1.782314	0.611924
1	2.385872	1.149653	-1.729900
1	2.357928	1.879230	-0.125622
1	2.248961	0.201439	2.243201
1	2.207524	-1.554255	2.079905
1	2.247758	-2.774664	-0.570521
1	2.312071	-1.750965	-2.007556
1	5.612128	-0.435803	-1.306551
1	4.193773	-0.295149	-2.358440
1	5.578469	0.563901	0.882489
1	4.134584	1.432599	1.427926
1	5.515399	-1.824472	0.645706
1	4.028846	-2.708893	1.029490
80	-0.778885	-0.157556	-0.045410
53	-1.035380	2.771331	0.018925
53	-3.081031	-1.736638	0.032506

The atomic coordinates (Å) of Hg^{2+}(PTA).

15	-0.493085	2.358316	-0.050185
6	0.385993	3.383063	-1.303675
6	-2.243052	2.772685	-0.450690
6	-0.243783	3.388449	1.456889
7	-0.112432	4.750611	-1.222652
7	-2.384247	4.223197	-0.485050
7	-0.656186	4.755335	1.162404
6	0.139897	5.350582	0.088103
6	-1.543730	4.832692	-1.516463
6	-2.073627	4.836281	0.807050
1	1.457709	3.353272	-1.106670
1	0.209540	2.968379	-2.296291
1	-2.505041	2.338811	-1.415824
1	-2.897737	2.341498	0.306670
1	-0.834587	2.977791	2.275919
1	0.807099	3.358093	1.745221
1	-0.114311	6.409287	0.027844
1	1.199257	5.260845	0.329455
1	-1.812038	5.887125	-1.589527
1	-1.744523	4.355870	-2.475990
1	-2.348029	5.890322	0.754354
1	-2.669792	4.360657	1.586073
80	0.084688	0.062876	0.085683
